# Genome of *Phyllanthus emblica*: the medicinal plant Amla with super antioxidant properties

**DOI:** 10.3389/fpls.2023.1210078

**Published:** 2023-09-01

**Authors:** Shruti Mahajan, Manohar S. Bisht, Abhisek Chakraborty, Vineet K. Sharma

**Affiliations:** MetaBioSys Group, Department of Biological Sciences, Indian Institute of Science Education and Research Bhopal, Bhopal, Madhya Pradesh, India

**Keywords:** *Phyllanthus emblica*, amla, medicinal plant, genome sequencing, antioxidant, vitamin C biosynthesis

## Abstract

*Phyllanthus emblica* or Indian gooseberry, commonly known as amla, is an important medicinal horticultural plant used in traditional and modern medicines. It bears stone fruits with immense antioxidant properties due to being one of the richest natural sources of vitamin C and numerous flavonoids. This study presents the first genome sequencing of this species performed using 10x Genomics and Oxford Nanopore Technology. The draft genome assembly was 519 Mbp in size and consisted of 4,384 contigs, N50 of 597 Kbp, 98.4% BUSCO score, and 37,858 coding sequences. This study also reports the genome-wide phylogeny of this species with 26 other plant species that resolved the phylogenetic position of *P. emblica*. The presence of three ascorbate biosynthesis pathways including L-galactose, galacturonate, and myo-inositol pathways was confirmed in this genome. A comprehensive comparative evolutionary genomic analysis including gene family expansion/contraction and identification of multiple signatures of adaptive evolution provided evolutionary insights into ascorbate and flavonoid biosynthesis pathways and stone fruit formation through lignin biosynthesis. The availability of this genome will be beneficial for its horticultural, medicinal, dietary, and cosmetic applications and will also help in comparative genomics analysis studies.

## Introduction

Vitamin C, also known as ascorbic acid, is a vital vitamin due to its multifaceted roles in animals as well as plants, and is an essential component of the human diet (Gallie, 2013; Carr and Maggini, 2017). The prolonged deficiency of this vitamin causes scurvy which was infamously responsible for killing thousands of sailors in the medieval period, since humans and primates cannot synthesise vitamin C due to the absence of an enzyme gulono-lactone oxidase (GULO), which is responsible for the final conversion to ascorbic acid (Martini, 2003; Wheeler et al., 2015). Thus, they depend majorly on plants that are the dominant sources of vitamin C for animals (Wheeler et al., 2015).

*Phyllanthus emblica* is one of the richest sources of natural vitamin C, and is, also known as Indian gooseberry or amla. It is an economically important medicinal horticultural plant that belongs to the family Phyllanthaceae in order Malpighiales, and is widely used in pharmaceuticals, nutraceuticals, food industry, and cosmetics sectors with an estimated market of USD 49.34 billion by 2025 (Muzaffar et al., 2022). Genus *Phyllanthus* is the largest genus of its family with approximately 1,000 species of which several are used as ethnomedicinal herbs due to the presence of medicinal phytochemicals (Sarin et al., 2014; Mao et al., 2016; Geethangili and Ding, 2018). The morphological characteristics of *P. emblica* include a light grey stem with thin flaky bark, simple leaves, and greenish-yellow unisexual flowers that are arranged like female flowers on the top and male flowers on the lower side. The fruits are typical drupes of about 2 cm in diameter, also known as stone fruits, with seeds encased in a hard lignified endocarp known as stone that helps in seed protection and dispersal (Dardick and Callahan, 2014; Dasaroju and Gottumukkala, 2014).

The geographical distribution of this prominent ethnomedicinal herbal species spreads from tropical to warm temperate regions like India, China, Sri Lanka, Bangladesh, Indonesia, Thailand, etc., among which India is the top producers of amla with annual production of 1,275,660 metric tonnes (Mao et al., 2016; Department of Agriculture & Farmers Welfare et al., 2021). This plant has also been used in many traditional medicine systems like Indian Ayurveda, Traditional Chinese Medicine System, etc. and is now widely used in modern medicines (Mao et al., 2016; Gul et al., 2022). Extracts of almost all parts of this plant such as leaf, bark, seed, root and fruit show medicinal properties like anti-microbial, anti-viral, anti-inflammatory, anti-oxidant, anti-aging, anti-diabetic, hypolipidemic, hypoglycaemic, neuroprotective, anti-cancer, immunomodulatory and hepatoprotective, etc. due to the presence of various secondary metabolites (phytochemicals) like alkaloids, phenolic acids, hydrolysable tannins, flavonoids, etc. with significance to human health and diseases (Gantait et al., 2021; Gul et al., 2022; Saini et al., 2022; Yan et al., 2022). The clinical effectiveness of *P. emblica* has been confirmed in diseases like dyslipidaemia, type 2 diabetes, chronic periodontitis, symptomatic knee osteoarthritis, etc. (Gantait et al., 2021). Amla is used in treating COVID-19 patients where its consumption shortened the recovery time (Varnasseri et al., 2022). Additionally, its phytochemicals are reported as potential protease inhibitors of SARS-CoV-2 virus through *in-silico* evidences (Murugesan et al., 2021; Pandey et al., 2021). Its extracts are proven to have protective effects by maintaining gut microbiome homeostasis *in vivo* (Li X. et al., 2022; Luo et al., 2022). Along with its benefits in human health, it is also effective in aquaculture, dairy and poultry as feed additives (Nguse et al., 2022; Van Doan et al., 2022; Abo Ghanima et al., 2023).

Among the vitamin C-rich fruits, *P. emblica* is known to contain the highest content of vitamin C (up to 720mg/100g fruit) along with other phytochemicals, minerals and amino acids (Kubola et al., 2011; Chavhan, 2017; Abeysuriya et al., 2020; Gul et al., 2022). Plants produce this vitamin to protect them against biotic (pathogens) and abiotic stresses (heat or light), and is also needed for the biosynthesis of plant hormones, and plant pigments, and acts as a cofactor in the cell cycle and metabolism, etc. (Gallie, 2013). The ascorbate biosynthesis occurs in plants through four proposed pathways i.e., L-galactose (also known as Smirnoff-Wheeler pathway), galacturonate (uronic acid pathway), L-gulose and myo-inositol pathways (Fenech et al., 2019; Paciolla et al., 2019). Among these pathways, the Smirnoff-Wheeler (SW) pathway is considered as the most common pathway of ascorbate biosynthesis (Gómez-García and Ochoa-Alejo, 2016; Sodeyama et al., 2021). Various genome-wide studies revealed ascorbic acid biosynthesis pathways in *Psidium guajava*, *Citrus sinensis*, kiwifruits, etc., however the ascorbate biosynthesis pathways have not been examined in *P. emblica* (Xu et al., 2013; Feng et al., 2021; Liao et al., 2021; Han et al., 2022).

Despite being a pharmaceutically and nutritionally important plant, the genome sequence of *P. emblica* still remains unknown. However, transcriptome studies were carried out previously to explore a few biosynthesis pathways in *P. emblica* (Kumar et al., 2016; Xiong-fang et al., 2018). The number of chromosomes in *P. emblica* was first reported as 28 in 1943 (Perry, 1943). Several following studies reported the chromosome numbers ranging from 52 to 104, and the most recent study has reported the presence of 100 chromosomes in *P. emblica* (Ammal and Raghavan, 1958; Soontornchainaksaeng and Chaiyasut, 1999; Rahman et al., 2021). Thus, to gain genomic insights into the medicinal properties of *P. emblica*, we performed its genome sequencing and assembly using a hybrid approach that includes 10x Genomics and Oxford Nanopore Technology (ONT) long-read sequencing technologies along with transcriptomic sequencing using the Illumina technology. Further, we analysed the genes involved in vitamin C, lignin and flavonoid biosynthesis pathways. We also constructed a genome-wide phylogenetic tree of *P. emblica* with 26 plant species, which were further analysed for gene family expansion and contraction. Furthermore, this study performed a comprehensive comparative evolutionary genomic analysis across 19 plant species to uncover the genes with multiple signatures of adaptive evolution in *P. emblica*.

## Materials and methods

### DNA-RNA extraction, species identification and sequencing

The leaves sample from an individual plant located at the campus of Indian Institute of Science Education and Research Bhopal, India (23.2858° N, 77.2755° E) were used in this study (**Supplementary Figure 1**). The DNA was extracted from the leaves sample using Carlson lysis buffer except for the precipitation step that was carried out with 0.5X volume of NaCl ad 0.7X volume of isopropanol (Jaiswal et al., 2021). The extracted DNA was quantified, and quality was checked on Qubit 2.0 fluorometer and Nanodrop 8000 spectrophotometer, respectively. Species identification assay was performed using marker genes: Internal Transcribed Spacer (*ITS*) and Maturase K (*MatK*). The extracted DNA was utilised to prepare libraries for 10x Genomics and nanopore sequencing that were sequenced on Illumina NovaSeq 6000 and MinION Mk1C sequencers, respectively. The RNA extraction from leaf tissue was performed as per the protocol described by Kumar and Singh (2012) with a few modifications (Kumar and Singh, 2012). The RNA was used for preparing the library using TruSeq Stranded Total RNA Library Preparation kit (Illumina Inc., CA, USA) with Ribo-zero Plant workflow and sequenced on Illumina NovaSeq 6000 instrument for generating 150 bp paired-end reads. The detailed method of DNA and RNA extraction with library preparation and sequencing is discussed in **Supplementary Text 1**.

### Genome assembly

The proc10xG set of python scripts (https://github.com/ucdavis-bioinformatics/proc10xG) was used to pre-process the 10x Genomics raw reads by removing the barcode sequences. The obtained reads were processed by SGA-preqc (paired-end mode) for genome size estimation which works on a k-mer distribution-based approach (Simpson and Durbin, 2012). For genome complexity assessment, these pre-processed reads were used by Jellyfish v2.2.10 and GenomeScope v2.0 for generating k-mer count histograms and calculating heterozygosity, respectively (Marçais and Kingsford, 2011; Ranallo-Benavidez et al., 2020).

Guppy v3.2.1 (Oxford Nanopore Technologies) was used to carry out base calling of nanopore raw reads. Adaptor removal was performed on this base called raw data using Porechop v0.2.4 (Oxford Nanopore Technologies). The pre-processed reads were utilised for genome assembly using three different assemblers: wtdbg v2.0.0, SMARTdenovo (https://github.com/ruanjue/smartdenovo), and Flye v2.9 (Kolmogorov et al., 2019; Ruan and Li, 2020). wtdbg v2.0.0 and Flye v2.9 were used with default settings whereas SMARTdenovo was used with zero as minimum read length (Kolmogorov et al., 2019; Ruan and Li, 2020). Quast v5.0.2 was employed to assess the genome assembly statistics (Gurevich et al., 2013). The genome assembly resulting from Flye v2.9 was considered for further analysis due to its better assembly statistics and assembled genome size (Kolmogorov et al., 2019). The assembly was polished three times by Pilon v1.24 using filtered reads. ARCS v1.2.2 and LINKS v2.0.0 (default settings) were employed for the first round of scaffolding using Longranger basic v2.2.2 (https://support.10xgenomics.com/genome-exome/software/pipelines/latest/installation) barcode filtered 10x Genomics linked reads and adaptor-removed nanopore reads, respectively (Walker et al., 2014; Warren et al., 2015; Yeo et al., 2018). After scaffolding, the quality-filtering of RNA-Seq paired-end raw reads was performed using Trimmomatic v0.39 with parameters- “LEADING:15 TRAILING:15 SLIDINGWINDOW:4:15 MINLEN:50” which were subsequently utilised by AGOUTI v0.3.3 for the second round of scaffolding (Bolger et al., 2014; Zhang et al., 2016).

Supernova v2.1.1 was used to perform *de novo* assembly of *P. emblica* with maxreads=all options with other default parameters (Weisenfeld et al., 2017). The obtained genome assembly was corrected by Tigmint v1.2.6 using Longranger basic v2.2.2 processed linked reads (Jackman et al., 2018). Further, the first round of scaffolding was performed with ARCS v1.2.2 and LINKS v2.0.0 using Longranger basic v2.2.2 processed linked reads and adaptor removed nanopore reads, respectively (Warren et al., 2015; Yeo et al., 2018). To increase the assembly contiguity, AGOUTI v0.3.3 was used with the pre-processed transcriptomic paired-end reads (quality-filtered) (Zhang et al., 2016).

RagTag v2.1.0 was used to merge both the assemblies obtained from Supernova and Flye assemblers using the patch command line utility (Alonge et al., 2021). RagTag uses the main assembly as reference and query assembly to fill the gap in the reference assembly. LR_Gapcloser was used to perform gap-closing of the assembly using pre-processed nanopore reads (Xu et al., 2019). Sealer v2.3.5 was used for gap-closing of the assembly using barcode-removed linked reads with 30-120 k-mer value and 10 bp interval (Paulino et al., 2015). The fixation of small indels, base errors, and local misassemblies was performed by Pilon v1.24 using pre-processed linked reads to provide the draft genome assembly of *P. emblica* (Walker et al., 2014). Obtained draft genome assembly was further length base filtered and scaffolds having length ≥5 kbp were retained. BUSCO v5.2.2 with embryophyta_odb10 single-copy orthologs dataset was employed to assess the completeness of genome assembly (Simão et al., 2015). For further assessment of the assembly quality, the barcode-filtered 10x Genomics reads, nanopore long reads and the quality-filtered transcriptomic reads were mapped onto the genome assembly using BWA-MEM v0.7.17, MiniMap2 v2.17 and HISAT2 v2.2.1, respectively, and SAMtools v1.13 “flagstat” was used to calculate the percentage of mapped reads (Li et al., 2009; Li, 2013; Kim et al., 2015; Li, 2018).

### Annotation of genome and construction of gene set

For annotation of repeats in the final genome assembly, RepeatModeler v2.0.3 was used to generate a *de novo* repeat library (Flynn et al., 2020). The clustering of repeats was performed using CD-HIT-EST v4.8.1 with parameters - 8 bp seed size and 90% sequence identity to eliminate redundant sequences (Fu et al., 2012). The resultant repeat library was utilised by RepeatMasker v4.1.2 (http://www.repeatmasker.org) to soft-mask the final genome assembly of *P. emblica*.

The coding gene set was constructed on the resultant repeat-masked genome assembly using MAKER v3.01.04 pipeline which deploys approaches such as *ab initio* and evidence alignment for prediction (Campbell et al., 2014). The construction of *de novo* transcriptome assembly was performed by Trinity v2.14.0 (default parameters) using quality-filtered transcriptomic reads of *P. emblica* from this study and previously reported studies (Haas et al., 2013; Liu et al., 2018). The gene set was constructed with transcriptome assembly and protein sequences of species belonging to the same order Malpighiales (*Populus trichocarpa* and *Manihot esculenta*) that were used as EST and protein evidence, respectively. In the MAKER pipeline, AUGUSTUS v3.2.3 was used for *ab initio* gene prediction while empirical evidence alignments and alignment polishing were performed using BLAST and Exonerate v2.2.0 (https://github.com/nathanweeks/exonerate), respectively (Altschul et al., 1990; Stanke et al., 2006). Based on the length and Annotation Edit Distance (AED) of gene models, the final gene set was constructed by selecting genes with length (≥150bp) and AED values <0.5. The completeness of this final gene set (also termed a high-confidence gene set) was checked using BUSCO v5.2.2 with embryophyta_odb10 dataset (Simão et al., 2015).

Additionally, Barrnap v0.9 (https://github.com/tseemann/barrnap) and tRNAscan-SE v2.0.9 were used to perform *de novo* prediction of rRNA and tRNA, respectively (Chan and Lowe, 2019). Based on homology, miRNA gene sequences in the *P. emblica* genome were identified using miRbase database with e-value 10^-9^ and 80% identity (Griffiths-Jones et al., 2007).

### Phylogenetic tree construction

The 26 plant species were selected from Ensembl plant release 54 for phylogenetic analysis considering the representation of each plant family among the selected species (Bolser et al., 2016). Besides the protein sequences of these selected 26 plant species, MAKER-derived protein sequences of *P. emblica* were used for phylogenetic tree construction. Among all the protein files, the longest isoform for each protein was selected and provided to OrthoFinder v2.5.4 to construct the set of orthologous genes (Emms and Kelly, 2019). KinFin v1.0 was used to extract fuzzy one-to-one orthologs protein sequences that were present in all 27 species (Laetsch and Blaxter, 2017). MAFFT v7.310 was used to individually align all the obtained fuzzy one-to-one orthologs which were filtered and concatenated using BeforePhylo v0.9.0 (https://github.com/qiyunzhu/BeforePhylo) (Katoh and Standley, 2013). These obtained protein sequences were used to construct a phylogenetic tree based on maximum likelihood using RAxML with the ‘PROTGAMMAAUTO’ amino acid substitution model and 100 bootstrap values (Stamatakis, 2014).

Amino acid sequences of *MatK* gene from 49 *Phyllanthus* species (top 49 species except *P. emblica* based on sequence length) and *Zea mays* (outgroup) obtained from UniProt database along with MatK protein sequence of *P. emblica* were used for the phylogenetic analysis. MAFFT v.7.310 was used to align these protein sequences, and RAxML v8.2.12 was used with 1000 bootstrap value and ‘PROTGAMMAAUTO’ amino acid substitution model to construct the *MatK*-based phylogenetic tree (Katoh and Standley, 2013; Stamatakis, 2014).

### Gene family expansion and contraction analysis

The proteome files containing the longest isoform for every protein from selected 27 species along with generated species phylogenetic tree were provided to CAFE v5 to assess the evolution of gene families (Mendes et al., 2020). The species phylogenetic tree was adjusted to an ultrametric tree based on the calibration point of 120 million years between *P. emblica* and *Beta vulgaris* obtained from TimeTree database v5.0 (Kumar et al., 2022). BLASTP was performed on protein sequences of all 27 species in All-versus-All mode (Altschul et al., 1990). The BLASTP results were clustered using MCL v14-137 and gene families containing clade-specific genes and >100 gene copies for minimum of one species were eliminated. These resultant gene families and ultrametric species tree were used to analyse the evolution (expansion/contraction) of gene families using a two-lambda (λ) model where λ signifies a random birth-death parameter. Among the obtained contracted/expanded gene families, gene families with >10 genes were considered as highly contracted/expanded gene families.

### Identification of signatures of adaptive evolution

The 19 plant species were selected for identification of genes with evolutionary signatures that included five species of order Malpighiales i.e., *Linum usitatissimum, Manihot esculenta, Phyllanthus emblica*, *Populus trichocarpa* and *Ricinus communis* along with *Actinidia chinensis* (order Ericales)*, Arabidopsis thaliana* (order Brassicales)*, Coffea canephora* (order Gentianales)*, Cucumis sativus* (order Cucurbitales)*, Daucus carota* (order Apiales)*, Eucalyptus grandis* (order Myrtales)*, Ficus carica* (order Rosales)*, Gossypium raimondii* (order Malvales)*, Helianthus annuus* (order Asterales)*, Olea europaea* (order Lamiales)*, Pistacia vera* (order Sapindales)*, Quercus lobata* (order Fagales)*, Solanum lycopersicum* (order Solanales) and *Vitis vinifera* (order Vitales). Orthologous gene sets were constructed by OrthoFinder v2.5.4 using the proteome files from 19 selected plant species. Orthogroups that contained protein sequences from all these selected species were retrieved and in case multiple protein sequences were present for a species, the longest isoform of that protein was selected and retained for further analysis.

### Identification of genes with higher rate of evolution

The resulting orthogroups across 19 plant species were aligned individually using MAFFT v7.310 (Katoh and Standley, 2013). These obtained alignments were used to construct a phylogenetic tree for individual orthogroups using RAxML v8.2.12 with ‘PROTGAMMAAUTO’ amino acid substitution model and a bootstrap value of 100 (Stamatakis, 2014). R package “adephylo” was used to calculate root-to-tip branch length distance for genes of all species in the phylogenetic trees (Jombart et al., 2010). The genes of *P. emblica* with comparatively higher root-to-tip branch length distance values were extracted and listed as the genes with higher nucleotide divergence or rate of evolution.

### Identification of *P. emblica* genes with unique amino acid substitutions

Using the multiple sequence alignments obtained from MAFFT v7.310, which were used for the identification of genes with a high rate of evolution, amino acid positions alike in all the species except *P. emblica* were extracted and labelled as genes with unique amino acid substitution. Ten amino acids around any gap were not included in this analysis. The functional impact of obtained genes showing amino acid substitution was evaluated using Sorting Intolerant From Tolerant (SIFT) with UniProt database (Ng and Henikoff, 2003).

### Identification of positively selected genes

MAFFT v7.310 was used for individual alignment of nucleotide sequence of all orthologous gene sets across selected 19 species (Katoh and Standley, 2013). PAML v4.9a with “codeml” program based on a branch-site model used nucleotide alignments in PHYLIP format and a species phylogenetic tree of these 19 species (constructed using RAxML) to identify genes with positive selection (Yang, 2007). These obtained genes with their log likelihood values were further processed through likelihood-ratio tests and genes with FDR-corrected p-values of <0.05 were labelled as positively selected genes. Positively selected codon sites were identified using Bayes Empirical Bayes (BEB) analysis with criteria of >95% probability for the foreground lineage.

### Genes with multiple signatures of adaptive evolution (MSA)

The high rate of evolution, unique amino acid substitution with functional impact and positive selection are the evolutionary signatures of adaptive evolution. *P. emblica* genes that showed at least two of these evolutionary signatures were considered as the genes with multiple signatures of adaptive evolution (MSA).

### Functional annotation

The annotation of high-confidence gene sets of *P. emblica* was performed using NCBI non-redundant (nr) database, SWISS-PROT database and Pfam-A v32.0 database using BLASTP (10^-5^ e-value), BLASTP (10^-5^ e-value), and HMMER v3.3, respectively (Bairoch and Apweiler, 2000; Bateman et al., 2004; Finn et al., 2011). The coding genes including the genes with evolutionary signatures were functionally annotated using KAAS and eggNOG mapper (Moriya et al., 2007; Huerta-Cepas et al., 2017). Further, the considered contracted and expanded gene families of *P. emblica* were extracted and provided to KAAS v2.1 and eggNOG mapper v2.1.9 for functional annotation, respectively (Moriya et al., 2007; Huerta-Cepas et al., 2017). The functional annotation of contracted and expanded gene families was also checked manually on Kyoto Encyclopedia of Genes and Genomes (KEGG) database.

### Analysis of vitamin C biosynthesis genes

The protein sequences of all the available enzymes of all four proposed pathways of vitamin C biosynthesis for *A. thaliana* were downloaded from Swiss-Prot or NCBI database. The gene D-galactose reductase (*GalUR*) was not available for *A. thaliana*, thus, sequence from strawberry plant species was used. These protein sequences were matched against the protein sequences of *P. emblica* using BLASTP with e-value 10^-9^ (Altschul et al., 1990). The enzymes involved in galactose pathway from vitamin C-rich plants i.e., *Actinidia chinensis* (kiwi), *Capsicum annuum* (chilli pepper), *Carica papaya* (papaya), *Citrus sinensis* (sweet orange), *Malpighia glabra* (acerola), *Myrciaria dubia* (camu-camu), *Solanum lycopersicum* (tomato) and *Vitis vinifera* (grapes) along with *Arabidopsis thaliana* as an outgroup were also obtained from UniProt or NCBI databases. Six genes i.e., Mannose 1-phosphate guanylyl transferase (*GMPP*), GDP-D-Mannose 3’,5’-epimerase (*GME*), GDP-L-galactose phosphorylase (*GGP/VTC2/VTC5*) and L-galactose-1-phosphate phosphatase (*GPP/VTC4*), L-galactose dehydrogenase (*GalDH*) and L-galactono-1,4-lactone dehydrogenase (*GLDH*) were selected for phylogeny due to their sequence availability for all nine species. The phylogenetic tree was constructed using these six genes each from 10 selected species (including *P. emblica*) using RAxML (Stamatakis, 2014). Among the above expanded and contracted genes, we checked for the copy number of all the gene families involved in ascorbate biosynthesis and regeneration pathways in the *P. emblica* genome.

The distant orthologs of 20 genes involved in ascorbate biosynthesis and regeneration pathways were identified to elucidate their origin by HHblits web server with default parameters using UniRef30 database (Remmert et al., 2012). The top 10 hits considering unique genus for each gene were extracted and aligned using MAFFT v.7.310 (Katoh and Standley, 2013). These alignments were used to construct the phylogenetic trees for each of the genes using RAxML v8.2.12 (1000 bootstrap value and ‘PROTGAMMAAUTO’ amino acid substitution model) (Stamatakis, 2014).

### Analysis of flavonoid biosynthesis pathway

The protein sequences of genes involved in flavonoid biosynthesis pathway for *Manihot esculenta* were downloaded from UniProt and NCBI databases and matched against the protein sequences of *P. emblica* using BLASTP (e-value 10^-9^) (Altschul et al., 1990).

### Analysis of lignin biosynthesis pathway

The protein sequences of genes involved in lignin biosynthesis pathway for *Arabidopsis thaliana* were downloaded from UniProt and NCBI databases and matched against the protein sequences of *P. emblica* using BLASTP (e-value 10^-9^) (Altschul et al., 1990).

### Analysis of gene structure

Exonerate v2.2.0 was used to examine the exon-intron structure of genes involved in ascorbate biosynthesis and regeneration pathways, flavonoid biosynthesis, and lignin biosynthesis.

## Results

### Species identification and sequencing

The species was confirmed using the sequencing of two DNA markers, ITS and *MatK*, that were aligned to *P. emblica* sequences available at NCBI-nt database with the highest identity of 100% and 99.89%, respectively. A total of 136 Gbp (~237x coverage) and 18.3 Gbp (~32x coverage) of genome sequence data were generated using third-generation sequencing technologies i.e., 10x Genomics and Oxford Nanopore Technology (ONT), respectively (**Supplementary Table 1**). Further, ~85 million transcriptomic reads from leaf tissue were used for analysis in this study. (**Supplementary Table 2**).

### Genome assembly and annotation

We computationally estimated the genome size of *P. emblica* to be 579 Mbp. The final genome assembly had a size of 519 Mbp and consisted of 4,384 contigs with GC content of 33.49%, largest contig of 3.3 Mbp, and N50 of 597 Kbp (**Supplementary Table 3**). The heterozygosity was estimated to be 1.37%, which appears to be high given its small genome size. The 98.4% complete and 0.4% fragmented BUSCOs of this genome assembly indicated its completeness (**Supplementary Table 4**). Further, 96.8% of linked reads and 93.45% of nanopore long reads could be mapped on the final genome assembly. The repeats constituted 53.39% of the genome with 2,051 *de novo* repeat family sequences that were clustered into 1,803 repeat families. Among the interspersed repeats, 10.96% and 10.13% were predicted as Ty1/Copia and Gypsy/DIRS1 elements, respectively (**Supplementary Table 5**). A total of 815 transfer RNA (tRNA) and 141 ribosomal RNA (rRNA) genes were identified in the genome. The detailed information on 216 microRNAs (miRNA) of *P. emblica* genome is mentioned in **Supplementary Table 6**.

The *de novo* transcriptome assembly comprised of a total of 238,454 transcripts and these transcripts were used as EST (empirical evidence) in the MAKER pipeline. The high-confidence gene set constituted of 37,858 genes and had an 89.9% complete BUSCO score (**Supplementary Table 4**). Overall, ~ 96% (36,296 out of 37,858) high-confidence coding genes of *P. emblica* could be annotated using the three reference databases; NCBI-nr, Swiss-Prot, and Pfam-A (**Supplementary Table 7**). The functional annotations of coding genes of *P. emblica* are mentioned in **Supplementary Tables 8**–**10**.

### Phylogenetic tree construction

**Figure 1:**
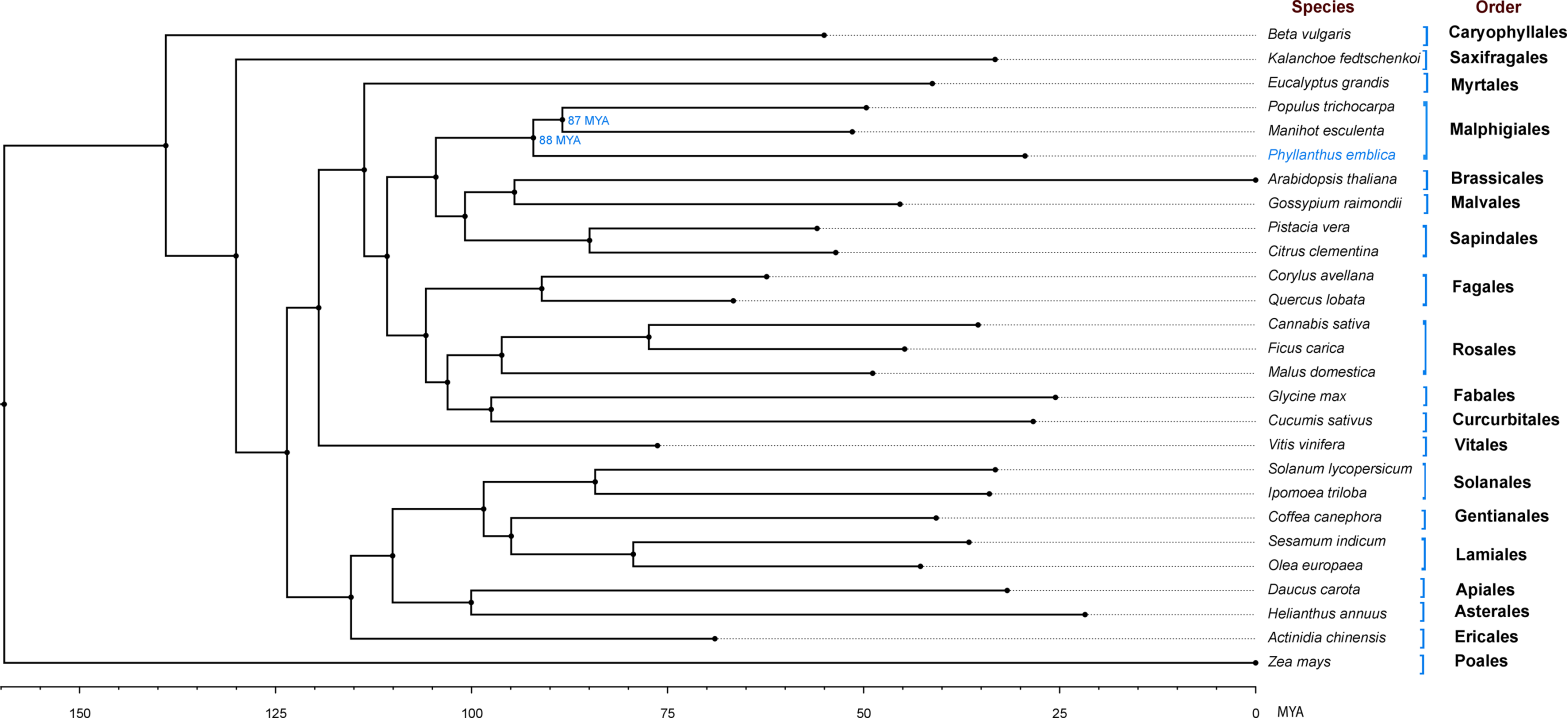
Genome-wide phylogeny of *P. emblica* with 26 other plant species Genome-wide phylogeny of *P. emblica* with 25 other eudicot species and a monocot species, *Zea mays* that was used as an outgroup. The indicated adjusted divergence time for Malpighiales order species were obtained from TimeTree database v5.0 (Kumar et al., 2022). The schematic representation method of the evolutionary time scale is similar to the previous studies (Teh et al., 2017; Xia et al., 2021).

**Figure 2:**
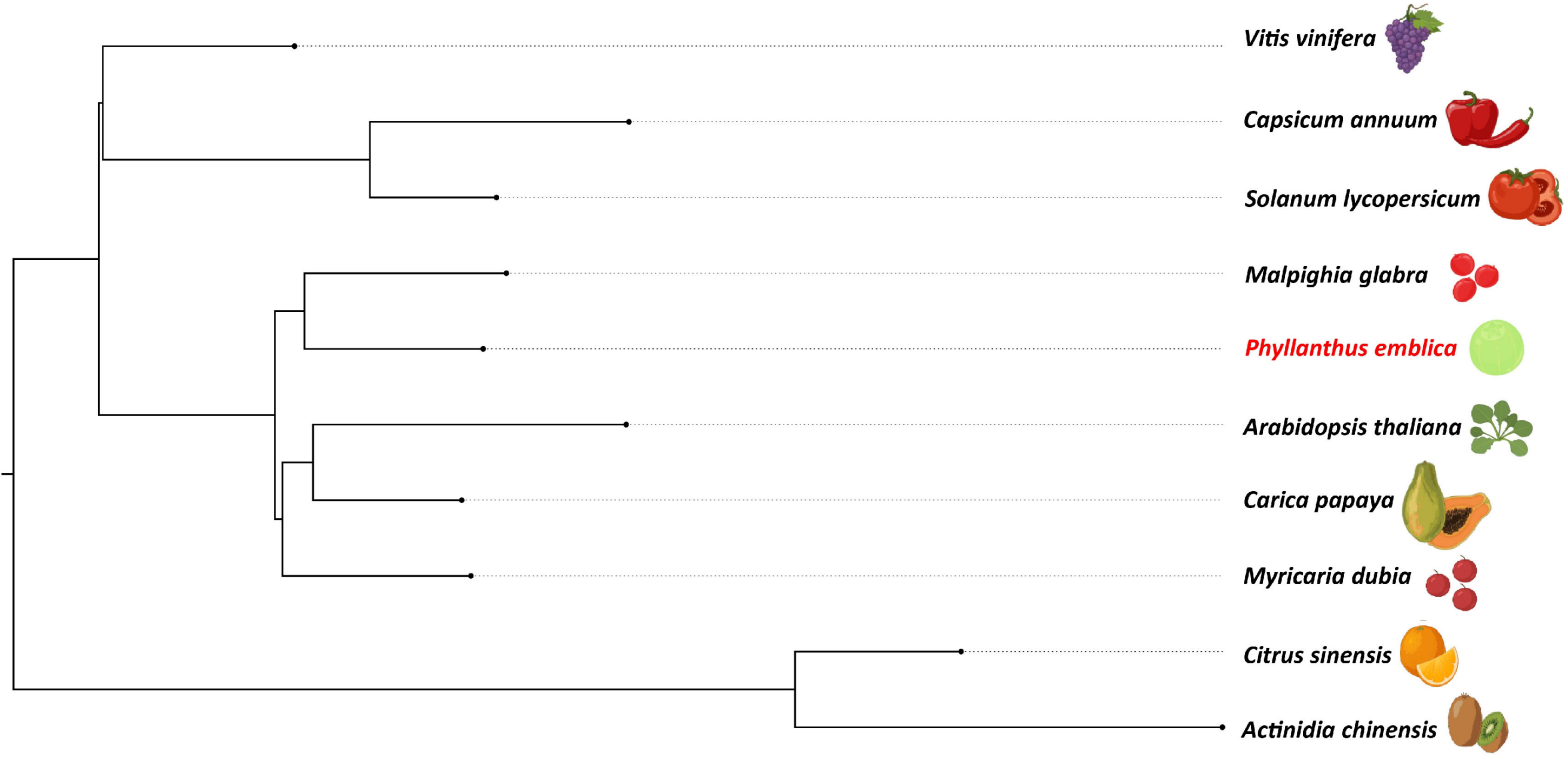
Vitamin C biosynthesis genes phylogeny of *P. emblica* with other vitamin C-rich fruits The phylogeny constructed for *P. emblica*, *Actinidia chinensis*, *Capsicum annuum*, *Carica papaya*, *Citrus sinensis*, *Myrciaria dubia*, *Malpighia glabra*, *Solanum lycopersicum*, *Vitis vinifera* and *Arabidopsis thaliana* using six genes of Smirnoff Wheeler pathway of ascorbate biosynthesis. The genes used were GDP-mannose pyrophosphorylase (*GMPP*), GDP-D-Mannose 3’,5’-epimerase (*GME*), GDP-L-galactose phosphorylase (*GPP/VTC2*), L-galactose-1-phosphate phosphatase (GPP/*VTC4*), L-galactose dehydrogenase (*GalDH*) and L-galactono-1,4-lactone dehydrogenase (*GLDH*).

We identified 145,194 orthogroups, of which 123 one-to-one fuzzy orthogroups were predicted from the selected 27 plant species. These selected concatenated protein sequence alignments of these fuzzy one-to-one orthogroups contained 104,108 alignment positions were used to construct a phylogenetic tree based on maximum likelihood using 26 eudicot species and *Zea mays* as an outgroup. The phylogenetic tree showed *Populus trichocarpa* and *Manihot esculenta* as the closest species to *P. emblica* as they belong to the same order Malpighiales. As per the phylogenetic tree, *P. emblica* diverged earlier (88 million years ago) than the other considered species of the order Malpighiales (**Figure 1** **;** **Supplementary Text 2**). Similarly, the phylogeny constructed with vitamin C biosynthesis genes followed the genome-wide phylogeny of *P. emblica* where the species like *P. emblica* and *Malpighia glabra*, *Arabidopsis thaliana* and *Carica papaya* and, *Capsicum annuum* and *Solanum lycopersicum* belonging to the same orders were sharing their common ancestral node (**Figure 2**). The phylogenetic tree of 50 *Phyllanthus* species using *MatK* indicated that *P. emblica* is evolutionarily closer to *P. urinaria* (**Supplementary Figure 2**), which has also been supported by other studies (Kathriarachchi et al., 2006; Bouman et al., 2021).

### Gene family expansion and contraction analysis

Gene family expansion and contraction analysis helps to identify the gene families that have increased or decreased in number in a given species. The analysis of adaptive evolution provides the clues of natural selection of specific phenotypic traits in a given species to cope with diverse environmental conditions that help in its survival. The gene expansion and contraction analysis showed a contraction of 1,048, and an expansion of 3,520 gene families in this species. Among these families, five and 42 gene families were found to be highly contracted and highly expanded, respectively. Among the 42 expanded gene families, 38 could be functionally annotated (**Supplementary Table 11**). The expanded gene families were majorly involved in lignin biosynthesis pathway, MAPK signaling pathway, transcription (as transcription factors), phenylpropanoid pathway, brassinosteroid biosynthesis, terpenoid biosynthesis, transportation (as transporters), plant hormone signal transduction, etc.

### Identification of signatures of adaptive evolution

For evolutionary analysis, 7,864 orthogroups were obtained across 19 selected species. Among these orthogroups, 46 genes showed higher nucleotide divergence and 488 genes showed positive selection in *P. emblica*. The genes of *P. emblica* were present in ~35% (2,791 of 7,864) of the orthogroups showed unique amino acid substitutions. A total of 236 genes were identified as MSA genes in *P. emblica*. The MSA genes were found to be involved in physiological processes like plant growth, ROS regulation and detoxification, DNA damage response, immune signaling, abiotic stress response, pathogen resistance, response to hormones like ethylene, abscisic acid, gibberellin and cytokinin, and cell wall modification. The list and functional details of the MSA genes of *P. emblica* are mentioned in **Supplementary Tables 12**, **13**.

### Vitamin C biosynthesis pathway

**Figure 3:**
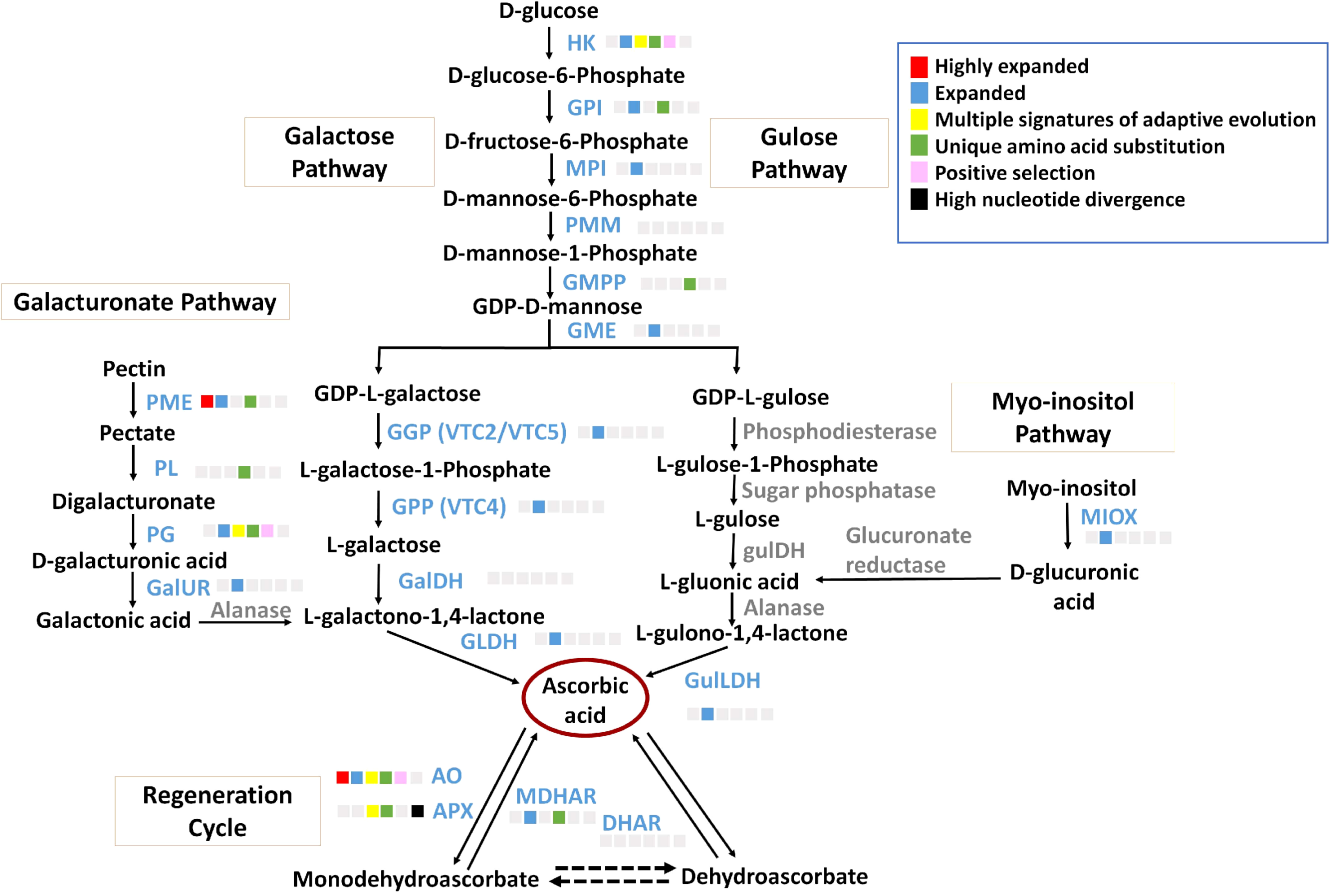
Ascorbate biosynthesis pathways The figure represents four proposed Ascorbate biosynthesis pathways i.e., Galactose pathway, Gulose pathway, Galacturonic acid pathway and Myo-inositol pathway and Ascorbate regeneration cycle. The enzymes of galactose pathway are HK, Hexokinase; GPI, Glucose 6-phosphate isomerase; MPI/PMI, Mannose 6-phosphate isomerase; PMM, Phosphomannomutase; GMPP, GDP-mannose pyrophosphorylase; GME, GDP-D-Mannose 3’,5’-epimerase; GGP/VTC2, GDP-L-galactose phosphorylase; GPP/VTC4, L-galactose-1-phosphate phosphatase; GalDH, L-galactose dehydrogenase; GLDH, L-galactono-1,4-lactone dehydrogenase. The Gulose pathway includes HK, GPI, MPI, PMM, GMPP, GME, Phosphodiesterase, Sugar phosphatase, L-gulose dehydrogenase, Aldonolactonase and Gulono-1,4-lactone dehydrogenase. The Galacturonic acid pathway includes PME, Pectin methylesterase; PL, Pectin lyase; PG, Polygalacturonse; GalUR, D-galacturonate reductase; Alanase, Aldono-lactonase; and GLDH. The Myo-inositol pathway includes MIOX, Myo-inositol oxidase; Glucuronate reductase; and GulLDH, Gulono-1,4-lactone dehydrogenase. The regeneration cycle of ascorbate includes AO, Ascorbate oxidase; APX, Ascorbate peroxidase; DHAR, Dehydroascorbate; and MDHAR, Monodehydroascorbate. The enzymes in blue colour and grey colour indicate presence and absence of their genes in *P.* emblica, respectively. The colour panel in front of each enzyme indicates the evolutionary signatures like highly expanded gene family, expanded gene family, MSA, unique amino acid substitution, positive selection and high nucleotide divergence shown by its gene in *P. emblica*.

Ascorbate, a non-enzymatic antioxidant, plays an important role in ROS detoxification and is a part of the ascorbate-glutathione pathway. *P. emblica* contains all the genes of the SW pathway similar to the other vitamin C-rich plant species like guava, kiwi, chilli pepper, etc., (Gómez-García and Ochoa-Alejo, 2016; Wang et al., 2018; Feng et al., 2021) (**Figure 3**). The gene structures of these genes are mentioned in **Supplementary Table 14**. The evolutionary analysis of six genes of SW pathway showed that *P. emblica* genes were phylogenetically closer to genes of *Malpighia glabra*, which also lies in the same order Malphigiales (**Figure 2**). Further, the genomic clues for presence of another pathway of ascorbate biosynthesis, i.e., galacturonate pathway were apparent from the presence of *PME*, *PL*, *PG*, *GalUR* and *GLDH* genes in *P. emblica* genome. This pathway is also proposed in tomatoes, strawberries, oranges, and grapes due to the presence of gene *GalUR*, which is the key gene of this pathway and was also present in *P. emblica* (Agius et al., 2003; Cruz-Rus et al., 2010; Cruz-Rus et al., 2011; Badejo et al., 2012; Xu et al., 2013). In addition, the genes *MIOX* and *GulLDH* involved in myo-inositol pathway were found, which supports the presence of the third pathway of ascorbate biosynthesis in *P. emblica*. However, the presence of the fourth pathway (L-gulose pathway) could not be confirmed due to lack of sufficient identification of genes involved in this pathway in *P. emblica* genome.

Among the genes of all proposed ascorbate biosynthesis pathways, six genes *HK*, *GPI*, *GMPP*, *PME*, *PL* and *PG* were identified with unique amino acid substitutions. Gene family of pectin methylesterase (*PME*) involved in galacturonate pathway of ascorbate biosynthesis was highly expanded. Other genes of the galacturonate pathway i.e., polygalacturonase (*PG*) was found with MSA, and pectin lyase (*PL*) gene had unique amino acid substitutions. Along with these biosynthesis genes, *MATE* (Multidrug And Toxic compound Extrusion) gene family, which is a vacuolar ascorbate transporter, was also highly expanded in *P. emblica* (Hoang et al., 2021). The details of ascorbate biosynthesis and regeneration pathways are described in **Figure 3** and **Supplementary Table 15**.

The phylogenetic relationships of 16 genes involved in ascorbate biosynthesis pathway with their distant orthologs are shown in **Supplementary Figures 3**–**18**. 10 out of 16 genes of ascorbate biosynthesis pathways had distant orthologs from other Malpighiales order members and among these genes, seven were phylogenetically closer to Malpighiales members. 11 genes had distant orthologs from monocot species. *GME* and *GPI* involved in the L-galactose pathway of ascorbate biosynthesis had algal and fungal orthologs, respectively. *GMPP* had orthology with algal and animal genes. *MPI* also had fungal and animal orthologs. *GalDH* and *PMM* had protozoan and animal orthologs along with a bacterial ortholog for *GalDH*. *GalUR* involved in the L-galacturonate pathway of ascorbate biosynthesis showed a fungal ortholog.

### Glutathione metabolism and ascorbate-glutathione pathway

Glutathione is another non-enzymatic antioxidant that plays a key role in different environmental stresses mainly oxidative stress and is a part of the ascorbate-glutathione pathway (Hasanuzzaman et al., 2019; Dorion et al., 2021). A total of four genes (*GPX*, *G6PD*, *gshA*, and *APX*) involved in glutathione metabolism showed multiple signatures of adaptive evolution and along with these genes, *GST* showed positive selection in *P. emblica*. The functional details of these genes are mentioned in **Supplementary Table 16**.

The ascorbate-glutathione pathway, also known as ascorbate regeneration cycle, plays an important role in oxidative stress response by converting the oxidised ascorbate forms to ascorbate and vice versa *via* four enzymes i.e., ascorbate oxidase (AO), ascorbate peroxidase (APX), dehydroascorbate reductase (DHAR) and monodehydroascorbate reductase (MDHAR) (Chen et al., 2003; Li et al., 2017). All these genes of ascorbate regeneration pathway were found in *P. emblica*. Among the four genes, *AO* and *APX* showed multiple signatures of adaptive evolution, and *MDHAR* showed unique amino acid substitutions. The functional details of these genes are mentioned in **Supplementary Table 16**. The phylogenetic relationships of these genes with their distant orthologs are shown in **Supplementary Figures 19**–**22**. Three genes involved in the ascorbate regeneration pathway had orthologs from bryophyte species (*APX* and *DHAR*) and algal species (*MDHAR*). Also, *DHAR* had a phylogenetically closer ortholog from Malpighiales order.

### Flavonoid biosynthesis pathway

**Figure 4:**
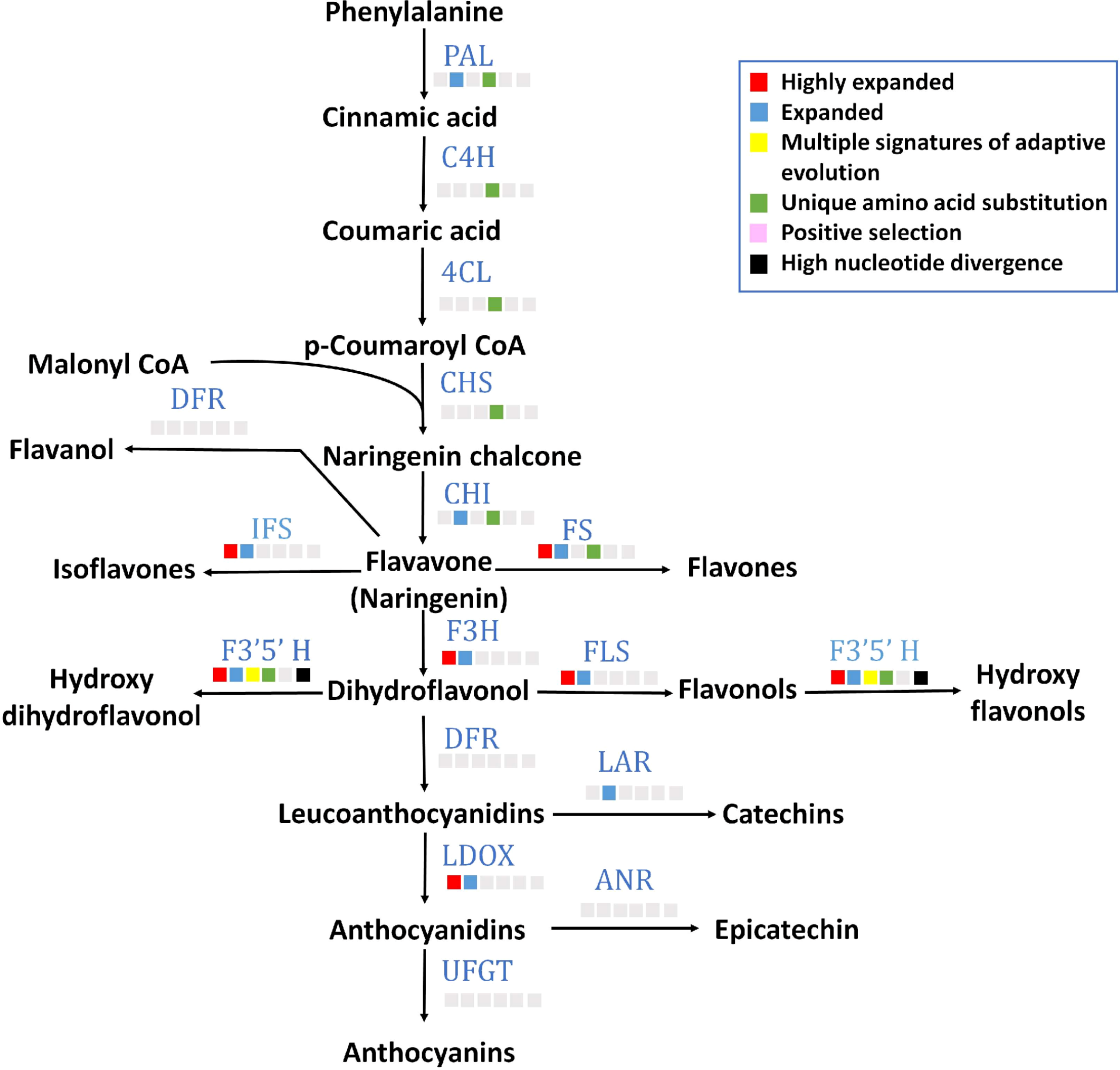
Flavonoid biosynthesis pathway The figure indicates the flavonoid biosynthesis pathway in *P. emblica*. The pathway includes enzymes PAL, phenylalanine ammonia lyase; C4H, cinnamate 4-hydroxylase;4CL, 4-coumaroyl CoA ligase; CHS, chalcone synthase; CHI, chalcone isomerase; FS, flavone synthase; DFR, dihydroflavonol 4-reductase; IFS, isoflavone synthase; F3’5’H, flavonol 3′5′ -hydroxylase; FLS, flavonol synthase; F3H, Flavanone 3-hydroxylase; LAR, leucoanthocyanidin reductase; LDOX, leucoanthocyanidin dioxygenase; ANR, anthocyanidin reductase; and UFGT, uridine diphosphate (UDP)-glucose flavonoid-3-O-glycosyltransferase. The blue colour of enzymes indicates presence of their genes in *P. emblica*. The colour panel in front of each enzyme indicates the evolutionary signatures like highly expanded gene family, expanded gene family, MSA, unique amino acid substitution, positive selection and high nucleotide divergence shown by its gene in *P. emblica*.

All 15 key genes of flavonoid biosynthesis pathway were found in *P. emblica* genome (**Figure 4**), and their gene structures are mentioned in **Supplementary Table 14**. Seven of these genes contained unique amino acid substitutions. Flavonoid 3’5’-hydroxylase (*F3’5’H*) was among the genes with MSA, and flavonol synthase (*FLS*), flavone synthase (*FS*), isoflavone synthase (*IFS*), flavavone 3-hydroxylase (*F3H*), leucoanthocyanidin reductase (*LDOX*) and *F3’5’H* gene families were highly expanded. The detailed pathway of flavonoid biosynthesis is showed in **Figure 4** and **Supplementary Table 17**.

### Lignified endocarp in this stone fruit

**Figure 5:**
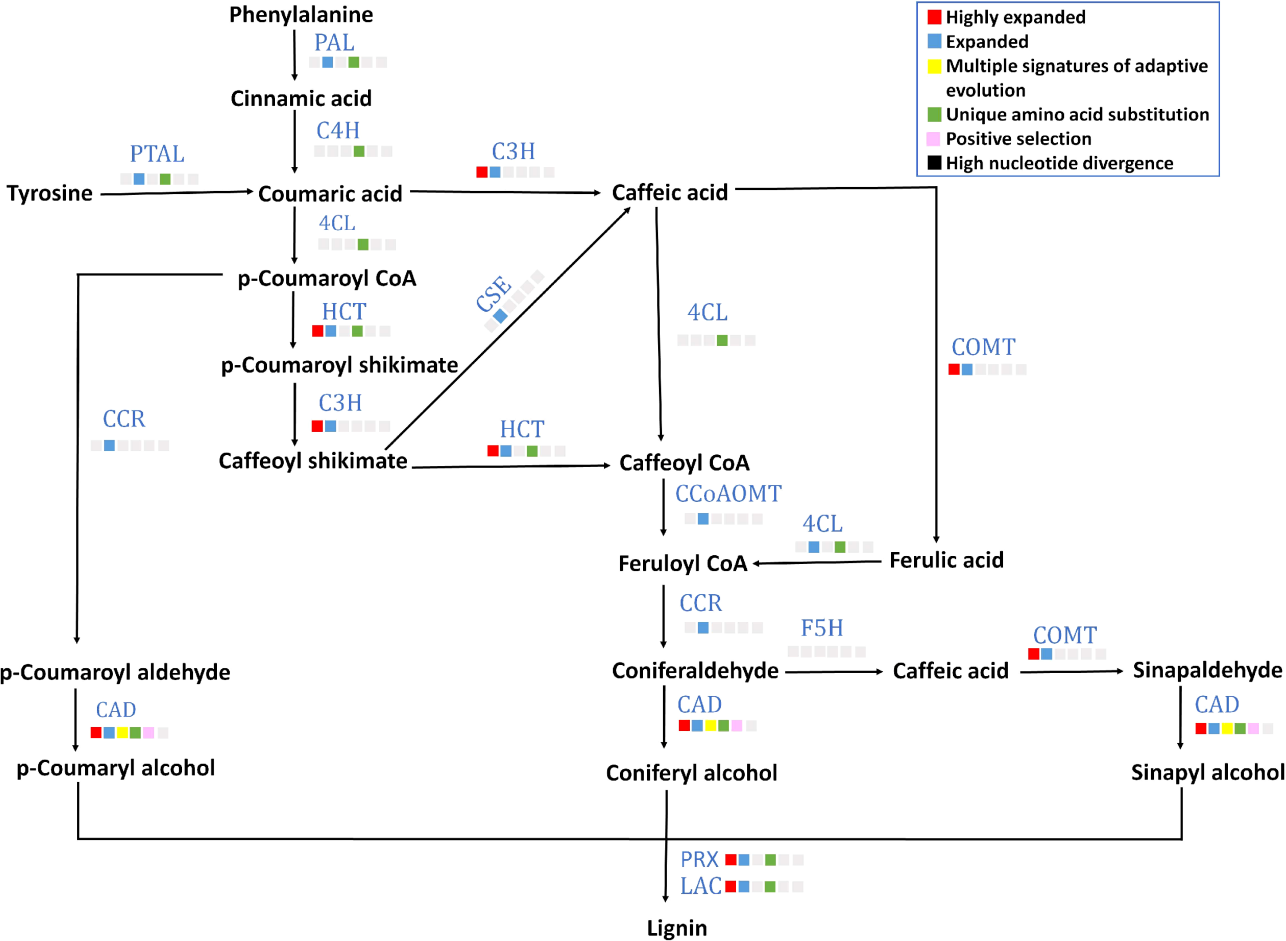
Lignin biosynthesis pathway The figure denotes lignin biosynthesis pathway in *P. emblica*. The pathway includes enzymes PAL, phenylalanine ammonia lyase; C4H, cinnamate 4-hydroxylase;4CL, 4-coumaroyl CoA ligase; PTAL, phenylalanine/tyrosine ammonia-lyase; C3H, 5-O-(4-coumaroyl)-D-quinate 3’-monooxygenase; HCT, shikimate O-hydroxycinnamoyl transferase; CSE, caffeoyl shikimate esterase; COMT, caffeic acid 3-O-methyltransferase; CCoAOMT, caffeoyl-CoA O-methyltransferase; CCR, cinnamoyl-CoA reductase; CAD, cinnamyl-alcohol dehydrogenase; F5H, ferulate-5-hydroxylase; PRX, peroxidase; and LAC, laccase. The blue colour of enzymes indicates presence of their genes in *P. emblica*. The colour panel in front of each enzyme indicates the evolutionary signatures like highly expanded gene family, expanded gene family, MSA, unique amino acid substitution, positive selection and high nucleotide divergence shown by its gene in *P. emblica*.

A lignified endocarp is a trait found in stone fruits like *P. emblica*. Lignin is important in stone cell formation in a drupe fruit that provides rigidity for seed protection and dispersal. Eight out of 13 genes (gene structures are mentioned in **Supplementary Table 14**) of lignin biosynthesis pathway including *PAL*, *4CL*, *C4H*, *PTAL*, *HCT*, *CAD*, *POD* and *LAC* contained unique amino acid substitutions (**Figure 5**). Among these eight genes, *CAD* showed multiple signatures of adaptive evolution. Furthermore, six gene families *C3H*, *HCT*, *COMT*, *CAD*, *POD* and *LAC* were highly expanded. Moreover, the gene families of transcription factors *MYB* and *LBD18* that are involved in lignin biosynthesis pathway were also found to be highly expanded (**Supplementary Table 18**).

### Plant growth, hormone and stress response

Among the 236 MSA genes, 36 genes were found to be involved in plant growth and development. These 36 genes are involved in cell division, flower development, seed development, seed germination, shoot development, cell elongation, sugar metabolism, cell wall biosynthesis, and root development, etc. (**Supplementary Table 19**). Phytohormones such as auxin, cytokinin, gibberellic acid, abscisic acid (ABA), ethylene, etc. help in plant growth, development, stress tolerance, etc. throughout the plant life. 20 MSA genes were responsible for plant hormone biosynthesis, signalling and response in plants. These include genes associated with plant hormone responses related to auxin, abscisic acid, ethylene, jasmonic acid, and cytokinin (**Supplementary Table 19**).

Plants have mechanisms for stress tolerance against abiotic and biotic stresses. 38 out of 236 MSA genes were associated with various responses against these stresses. Among these, 30 genes were associated with responses to abiotic stresses like salt, cold, heat, drought, etc., whereas 19 genes were associated with biotic stress tolerance. These genes are involved in stress responses like stress signal transduction, secondary metabolite biosynthesis, abscisic acid biosynthesis, ROS detoxification, stress-specific gene regulation, degradation of misfolded and damaged proteins, DNA damage response, cell wall modification, disease resistance, etc. (**Supplementary Table 19**). Also, six MSA genes were found to be involved in DNA damage repair mechanism in plants against environmental stresses (**Supplementary Table 19**).

### ROS regulation and detoxification

Reactive oxygen species (ROS) are metabolic by-products produced in mitochondria, plastids and peroxisomes, which can cause irreversible DNA damage resulting into cell death. In plants, ROS not only cause harmful effects but also act as signalling molecules for plant growth and stress responses. 18 out of 236 MSA genes were associated with ROS response, regulation and detoxification. These genes are involved in porphyrin biosynthesis, ROS-induced responses, ROS scavenging, biosynthesis and protecting antioxidant enzymes, maintaining homeostasis, accumulation, and biosynthesis of antioxidants, and activation of Fe-S cluster (**Supplementary Table 20**).

## Discussion

*P. emblica* or amla is a widely used medicinal plant with enormous antioxidant properties (Gul et al., 2022). To understand the genomic basis of these properties, we successfully constructed the first draft genome assembly of *P. emblica* using a hybrid sequencing approach using 10x Genomics, Illumina and ONT technologies. Despite a repetitive and highly heterozygous nature of this genome, implementation of a hybrid approach helped in constructing a high-quality genome assembly with N50 of ~0.6 Mbp and high BUSCO completeness (98.4%).

Further, this study is the first to resolve the genome-wide phylogenetic position of *P. emblica* with respect to 26 other plant species and found its early divergence from *Manihot esculenta* and *Populus trichocarpa* species of order Malpighiales, which was also confirmed by the adjusted time obtained from TimeTree database. Our phylogeny is also supported by the revised classification of order Malpighiales where Phyllanthaceae was separated as individual family from Euphorbiaceae in the Angiosperm Phylogeny Group Classification (APG III) (Group, 2009; Kawakita and Kato, 2017). The phylogeny of vitamin C biosynthesis genes also followed the genome-wide phylogeny. Phylogenetic analysis of genes involved in the L-galactose pathway of ascorbate biosynthesis showed that six out of 10 genes in *P. emblica* were closer to orthologs from other Malpighiales members. Further, key genomic insights were gained from the results of gene family expansion and contraction and from the genes with multiple signatures of adaptive evolution in *P. emblica*. The genes related to the biosynthesis of ascorbic acid, lignin and flavonoid were found to be evolutionarily selected in *P. emblica*.

Ascorbic acid is the major antioxidant in *P. emblica* and its fruit “amla” is one of the richest natural sources. A transcriptomic study of oranges had shown the attribution of different pathways of ascorbate biosynthesis in a tissue-specific as well as fruit developmental stage specific manner (Caruso et al., 2021). This could also be possible in *P. emblica* where the presence of three pathways of ascorbate biosynthesis is traced, and they could have roles in different stages and tissues. It was noted that the genes of one of the ascorbic acid biosynthesis pathways i.e., galacturonate pathway were found with MSA (*PG*), amino acid substitutions (*PME*, *PL* and *PG*) and highly expanded gene family (*PME*) in the *P. emblica* genome. The involvement of enzymes PME and PG in increased ascorbate production in tomatoes along with the role of PME in regulating ascorbate content through galacturonate pathway is shown in previous studies (Di Matteo et al., 2010; Badejo et al., 2012; Ruggieri et al., 2015; Rigano et al., 2018). Thus, it is tempting to speculate that the evolution of genes of galacturonate pathway could be associated with the high ascorbate production in *P. emblica*.

*P. emblica* is also rich in flavonoids that are synthesised in response to plant stress and contribute to its antioxidant property. The genes from *PAL* to *CHI* involved in the initial part of flavonoid biosynthesis pathway were found with unique amino acid substitutions, and the *F3’5’H* gene, which is previously reported to increase flavonoid accumulation, showed multiple signatures of adaptation (Wang et al., 2014; Nguyen et al., 2021). In addition to these, *FLS*, *F3’5’H*, *FS*, *LDOX*, *F3H* and *IFS* gene families were highly expanded which collectively indicates evolution of flavonoid biosynthesis genes in *P. emblica*. These genes are involved in biosynthesis of flavonoids such as isoflavones, flavones, anthocyanins and flavonols that have antioxidant properties and provide tolerance against various abiotic and biotic stresses (Verhoeyen et al., 2002; Agati et al., 2012). The evolutionary selection of these flavonoid associated genes might be responsible for the high antioxidant property and stress tolerance of *P. emblica*.

Being a stone fruit, a lignified endocarp is a trait found in the fruits of *P. emblica*, thus the evolution of lignin biosynthesis pathway was one of the major findings. Lignin is important for the stony seed coat formation in drupes that provides rigidity for its protection. The lignin biosynthesis genes were observed to be highly expanded and among the MSA genes in *P. emblica* genome, which hints towards the evolutionary significance of lignified endocarp in this stone fruit. The lignin biosynthesis gene families were also reported to be expanded in the other stone fruit genomes such as pear and *Populus*, which are economically important due to their fruit and wood, respectively, where lignin is the main content of pear’s stone cells and poplar’s wood (Wu et al., 2013; Li et al., 2022).

*P. emblica* also produces a large variety of secondary metabolites that provide tolerance against plant stresses. The expansion of gene families and MSA in genes involved in biosynthesis of various secondary metabolites and pathogen resistance against abiotic and biotic stresses were found that indicates the evolution of stress tolerance genes in this genome. Among the genes related to plant stress tolerance, the genes involved in ROS regulation and detoxification were also evolved in *P. emblica*.

Taken together, it is apparent that the adaptive evolution in genes involved in ascorbate biosynthesis, glutathione metabolism, flavonoid biosynthesis, and ROS detoxification are associated with the high antioxidant potential of *P. emblica*, which makes it a valuable herbal plant for use in traditional and modern medicine, horticulture, food and cosmetic products. Further, the high concentration of vitamin C in the amla fruit and the large production (up to 100 kg) of fruits per tree compared to other vitamin C rich fruits like *Malpighia glabra* (15-30 Kg/tree) and *Myrciaria dubia* (25-30 Kg/tree), makes it the perfect choice in switching from synthetic to natural supplementation of vitamin C (Rodrigues et al., 2001; Orwa et al., 2009; Carr and Vissers, 2013). Further, this plant also shows high genetic diversity and easy adaptation to various climatic zones and environmental conditions (Liu et al., 2020). The availability of the first draft genome of this economically important plant is likely to help in developing improved nutraceuticals, food, cosmetics and pharmaceutical products, and for further horticultural and genomic studies.

## Data availability statement

The datasets presented in this study can be found in online repositories. The names of the repository/repositories and accession number(s) can be found below: https://www.ncbi.nlm.nih.gov/, BioProject accession- PRJNA947813 and BioSample accession SAMN33867225.

## Author contributions

VKS conceived and coordinated the project. SM performed sample collection, DNA-RNA extraction, prepared samples for sequencing, performed long read sequencing, species identification assays, functional annotation of gene sets, and constructed all the figures. MSB and AC designed computational framework of the study, and performed all the computational analyses presented in the study. SM, MSB, AC and VKS analysed the data and interpreted the results. SM and VKS wrote the first draft of manuscript. SM, AC, MSB and VKS wrote and prepared the final manuscript. All the authors have read and approved the final version of the manuscript.
