# Supplementary material for: Genome of Phyllanthus emblica: the medicinal plant Amla with super antioxidant properties
Source: Front Plant Sci. 2023 Sep 1;14:1210078. doi: 10.3389/fpls.2023.1210078 (PMC10505619; doi:10.3389/fpls.2023.1210078)
Supplement: Supplementary file 1 [file DataSheet_1.pdf]

**Title: Genome of *Phyllanthus emblica*: the medicinal plant Amla with super antioxidant properties**

**Authors:** Shruti Mahajan[1], Manohar S. Bisht[1], Abhisek Chakraborty[1], Vineet K Sharma[1]*

**Affiliation:** [1]MetaBioSys Group, Department of Biological Sciences, Indian Institute of Science Education and Research Bhopal, Bhopal, 462066 Madhya Pradesh, India

*Correspondence: Vineet K Sharma - vineetks@iiserb.ac.in

**E-mail addresses of authors:** Shruti Mahajan – shruti17@iiserb.ac.in, Manohar S. Bisht – manohar21@iiserb.ac.in, Abhisek Chakraborty – abhisek18@iiserb.ac.in, Vineet K Sharma – vineetks@iiserb.ac.in

**Keywords:** *Phyllanthus emblica*, Amla, medicinal plant, genome sequencing, antioxidant, Vitamin C biosynthesis

**Supplementary Text 1**

**Experimental methods**

**DNA and RNA extraction**

The leaves sample from a plant located at the campus of Indian Institute of Science Education and Research Bhopal, India (23.2858° N, 77.2755° E) (**Supplementary Figure 1**). The 5g of fresh leaves (without the midribs) were collected, cleaned, and taken in an autoclaved and pre-chilled mortar pestle. The Carlson lysis buffer [100mM Tris-HCl, 2% Cetyl trimethyl ammonium bromide (CTAB), 1.4 M NaCl, 1% Polyethylene glycol (PEG) 8000 and 20mM Ethylenediamine tetraacetic acid (EDTA) (pH 9.5)] was pre-heated at 65˚C for 30 mins (Jaiswal, Mahajan et al. 2021). The 50 mL of pre-heated lysis buffer was added to the mortar pestle containing leaves and ground without liquid nitrogen until all the leaves were crushed. 100 µL of β-mercaptoethanol and 1 g of polyvinyl pyrrolidone 40 (PVP40) were added to it while grinding with lysis buffer. The homogenized leaves in lysis buffer were transferred to a 50 mL tube, added 100 µL of Proteinase K (20µg/mL) and 50 µL of RNase A (20mg/mL), and mixed by inverting tubes multiple times. The tube was incubated at 65˚C for 1 hr with intermittent mixing by inversion. After lysis of 1 hr, RNase treatment for 30 mins at 37˚C was given. The tube was then added with an equal volume of Chloroform: iso amyl alcohol (24:1) and centrifuged at 5,000xg for 15 mins. The aqueous layer was taken in a new 50 mL centrifuge tube, and again added with an equal volume of chloroform: isoamyl alcohol (24:1) and centrifuged at 5,000xg for 15 mins. The aqueous phase was collected in a 50 mL tube and precipitated with ½ volume of 0.5 M NaCl and 0.7 volume of isopropanol. The tubes were incubated at -20˚C overnight. Subsequently, the DNA was pellet down by centrifuging at 5,000xg for 45 mins. The DNA pellet was washed twice with 70% ethanol. The pellet was air-dried and resuspended in 100 µL of nuclease free water (NFW). The DNA quantity was calculated on Qubit 2.0 fluorometer using a qubit ds DNA Broad Range assay kit (Invitrogen, Thermofisher Scientific, USA). The DNA sample quality was checked on Nanodrop 8000 spectrophotometer. For Nanopore sequencing, highly pure samples with nanodrop ratios 260/280 and 260/230 as ~1.8 and 2.2 are recommended, therefore the DNA samples were purified with 0.45X Ampure XP magnetic beads (Beckman Coulter, Brea, CA) and DNA samples near to recommended nanodrop ratios were used for nanopore sequencing.

For RNA extraction from leaf tissue, the protocol described in Kumar and Singh (2012) was followed with a few modifications like overnight RNA precipitation step at -20˚C and final

elution in 30 µl of NFW (Kumar and Singh 2012). The extracted RNA was diluted to 1:10 dilution and quantified on Qubit 2.0 fluorometer using a qubit RNA high sensitivity assay kit (Invitrogen, Thermofisher Scientific, USA). The RNA was stored at -80˚C till library preparation.

**Species identification**

For species identification, two marker genes- Nuclear Internal Transcribed Spacer (ITS) and plastidal Maturase K (*MatK*) were used (Inglis, Mata et al. 2018). The Polymerase Chain Reaction (PCR) programs and primers used for the amplification are as follows:

1. ITS

   PCR program: Initial denaturation at 94˚C for 3 minutes,
   35 cycles of denaturation at 94˚C for 1 min, annealing at 55˚C for 1 min, and extension at 72 ˚C for 2.5 mins, and
   Final extension at 72 ˚C for 10 mins.

   Primers: Forward ⟶ 5′-TCCGTAGGTGAACCTGCGG-3′
   Reverse ⟶ 5′-TCCTCCGCTTATTGATATGC-3′

2. *MatK*

   PCR program: Initial denaturation at 95˚C for 3 mins,
   35 cycles of denaturation at 95˚C for 30 sec, annealing at 50˚C for 3 mins, and extension at 72 ˚C for 1.15 mins, and
   Final extension at 72 ˚C for 7 mins.

   Primers: Forward ⟶ 5’-CGATCTATTCATTCAATATTTC-3’
   Reverse ⟶ 5’-TCTAGCACACGAAAGTCGAAGT-3’

The *MatK* and ITS genes were amplified on veriti 96 well thermal cycler (Applied Biosystems, Thermofisher Scientific, USA) using enzymes Taq Polymerase (Invitrogen, Thermofisher Scientific, USA) and Paq polymerase (Agilent Technologies, Santa Clara, CA), respectively. The amplified products were checked on 2% agarose gel electrophoresis and the amplified products were purified using PureLink PCR purification kit using B2 buffer (Invitrogen, Thermofisher Scientific, USA). The purified amplicons were quantified on Qubit 2.0 fluorometer using a qubit ds DNA broad range assay kit (Invitrogen, Thermofisher Scientific,

USA). 20 ng of amplicons were sequenced on a sanger sequencer at Central Instrumental Facility (CIF), IISER Bhopal. The sequences were aligned to NCBI non redundant nucleotide database (nt) using blastn (Altschul, Gish et al. 1990). The species obtained from the blastn result with the highest identity is identified as the species.

**Library preparation and sequencing**

For genome sequencing, the DNA sample was taken for linked reads library preparation on chromium controller instrument using Gel Bead Kit v2 and Chromium Genome Library kit (10x Genomics, CA) by following the manufacturer's instructions (Chakraborty, Mahajan et al. 2021). The prepared library was checked on Agilent TapeStation using High Sensitivity D1000 screentape (Agilent Technologies, Santa Clara, CA) for quality and quantified on a Qubit fluorometer (Invitrogen, Thermofisher Scientific, USA). The prepared library was used for 150 bp paired-end sequencing on NovaSeq 6000 platform (Illumina Inc., USA). For long-read genomic sequencing, 1.5 µg of DNA sample having oxford nanopore technology recommended nanodrop ratios were used for library preparation using SQK-LSK110 library preparation kit (Oxford Nanopore Technologies, UK) by following manufacturer's instruction with a few modifications like using 1.5 µg of DNA and increasing the incubation time to 30 mins at adaptor ligation step. The library was loaded on flow cell FLO-MIN106 and sequenced on the MinION Mk1C platform (ONT, UK) using MinKNOW version 22.03.2.

For transcriptome sequencing, the RNA was checked on Nanodrop 8000 spectrophotometer, 1% agarose gel electrophoresis and TapeStation using High Sensitivity D1000 screentape (Agilent, Santa Clara, CA). From this total RNA, rRNA was removed by following the Ribo-Zero Plant workflow. The library was prepared using TruSeq Stranded Total RNA Library Preparation kit (Illumina Inc., CA, USA). The quality and quantity of RNA library were assessed on Tapestation 4150 using HS D1000 screentape (Agilent Technologies, Santa Clara, CA) and Qubit fluorometer using qubit ds DNA HS assay kit (Invitrogen, Thermofisher Scientific, USA), respectively. The transcriptomic library was sequenced on NovaSeq 6000 platform (Illumina Inc., USA) for 150 bp paired-end reads.

**Supplementary Text 2**

**Phylogeny related text**

Family Phyllanthaceae got separated as an individual plant family having two ovules in an ovary (one in each locule) as its distinguishable morphological character from Euphorbiaceae family members in Angiosperm Phylogeny Group classification (APG III) (Group 2009; Kawakita and Kato 2017). This has also been represented in our phylogeny as families Phyllanthaceae, Salicaceae and Euphorbiaceae were sharing same ancestral node, and Phyllanthaceae showed early divergence (Xi, Ruhfel et al. 2012). All the other plant species belonging to the same order are clustered together and shared the same ancestral node in the phylogeny, for example, all species of order Fagales, Lamiales, Malpighiales, Rosales, Sapindales, and Solanales shared their respective common nodes. Species of the same orders such as *Corylus avellana* and *Quercus lobata*, *Olea europaea* and *Sesamum indicum*, *Citrus clementina* and *Pistacia vera*, and, *Ipomoea triloba* and *Solanum lycopersicum* found to be present on the same branch in the phylogenetic tree. Members of Rosales order i.e., *Cannabis sativa*, *Ficus carica* and *Malus domestica* Golden were also found to share common node in the phylogeny which is also supported by other phylogenies (Ha, Shim et al. 2019; Mahajan, Chakraborty et al. 2021; Wang, Gu et al. 2021; Xu, Wu et al. 2021; Chakraborty, Mahajan et al. 2022).

**Supplementary Table 1. Genomic sequencing data details**

| Species | Total 10x Genomics reads | Total 10x Genomics data (bp) | Total Nanopore reads | Total Nanopore data (bp) |
|---|---|---|---|---|
| *Phyllanthus emblica* | 859,130,888 | 136,601,811,192 | 4,363,211 | 18,384,345,439 |

**Supplementary Table 2. Transcriptomic sequencing data details**

| Species | Total number of reads | Total number of bases (bp) | Source Tissue | Origin |
|---|---|---|---|---|
| *Phyllanthus emblica* | 8,538,890 | 1,374,761,290 | Leaf | Our study |
| *Phyllanthus emblica* | 39,933,248 | 5,991,900,473 | Leaf | Other study* |
| *Phyllanthus emblica* | 36,488,257 | 10,946,477,100 | Leaf | Other study (Liu, Ma et al. 2018) |

*Unpublished publicly available data at NCBI (SRR13512341)

**Supplementary Table 3. Statistical summary of genome assembly**

| Parameters | Values |
|---|---|
| Contigs (>= 0 bp) | 4,384 |
| Contigs (>= 1 Kbp) | 4,384 |
| Contigs (>= 5 Kbp) | 4,384 |
| Contigs (>= 10 Kbp) | 3,225 |
| Contigs (>= 25 Kbp) | 1,941 |
| Contigs (>= 50 Kbp) | 1,256 |
| Total length (bp) (>= 0 bp) | 519,191,968 |
| Total length (bp) (>= 1 Kbp) | 519,191,968 |
| Total length (bp) (>= 5 Kbp) | 519,191,968 |
| Total length (bp) (>= 10 Kbp) | 510,879,419 |
| Total length (bp) (>= 25 Kbp) | 490,207,416 |
| Total length (bp) (>= 50 Kbp) | 466,574,486 |
| Contigs | 4,384 |
| Largest contig (bp) | 3,342,314 |
| Total length (bp) | 519,191,968 |
| GC (%) | 33.49 |
| N50 (bp) | 596,556 |
| N75 (bp) | 237,796 |
| L50 | 249 |
| L75 | 591 |
| N's per 100 kbp | 6.07 |

**Supplementary Table 4. BUSCO statistics of *P. emblica* genome**

| Type of BUSCOs | Genome assembly | High confidence gene set |
|---|---|---|
| Complete | 1588 (98.4%) | 1452 (89.9%) |
| Fragmented | 7 (0.4%) | 91 (5.6%) |
| Missing | 19 (1.2%) | 71 (4.5%) |
| Total BUSCOs | 1614 | 1614 |

**Supplementary Table 5. Summary statistics of the *P. emblica* repetitive genomic regions detected by RepeatMasker**

| | | | | | |
|---|---|---|---|---|---|
| Total length: | 519,191,968 bp | | | | |
| GC (%) | 33.49% | | | | |
| Bases masked: | 277,188,201 bp (53.39%) | | | | |
| | | | Number of elements | Length occupied (bp) | Percentage of sequence |
| Retroelements | | | 103,495 | 113,571,390 | 21.87 % |
| | SINEs | | 0 | 0 | 0 % |
| | Penelope | | 0 | 0 | 0 % |
| | LINEs | | 3,276 | 1,615,106 | 0.31 % |
| | | CRE/SLACS | 0 | 0 | 0 % |
| | | L2/CR1/Rex | 0 | 0 | 0 % |

| | | | | | |
|---|---|---|---|---|---|
| | | R1/LOA/Joc key | 0 | 0 | 0 % |
| | | R2/R4/NeSL | 0 | 0 | 0 % |
| | | RTE/Bov-B | 704 | 233,729 | 0.05 % |
| | | L1/CIN4 | 2,572 | 1,381,377 | 0.27 % |
| | LTR elements: | | 100,219 | 111,956,284 | 21.56 % |
| | | BEL/Pao | 0 | 0 | 0 % |
| | | Ty1/Copia | 52,239 | 56,925,180 | 10.96 % |
| | | Gypsy/DIRS 1 | 45,422 | 52,613,314 | 10.13 % |
| | | Retroviral | 53 | 53,910 | 0.01 % |

**Supplementary Table 7. Information regarding coding gene annotation in *P. emblica***

| Database | Number of coding genes annotated |
|---|---|
| NCBI-nr | 36,180 (95.5%) |
| Swiss-Prot | 29,931 (79.0%) |
| Pfam-A | 29,433 (77.7%) |
| Total | 36,296 (~96%) |

**Supplementary Table 15. Genes involved in the Ascorbate biosynthesis pathway and ascorbate-regeneration pathway with copy number of their gene family**

| S.No. | Name | Symbol | Copy number |
|---|---|---|---|
| **Ascorbate biosynthesis pathway** | | | |
| 1 | Hexokinase | *HK* | 10 |
| 2 | Glucose 6-phosphate isomerase | *GPI* | 11 |
| 3 | Mannose 6-Phosphate isomerase | *PMI* | 4 |
| 4 | Phosphomannomutase | *PMM* | 2 |
| 5 | GDP-mannose pyrophosphorylase | *GMPP* | 3 |
| 6 | GDP-D-Mannose 3’,5’-epimerase | *GME* | 4 |
| 7 | GDP-L-galactose phosphorylase | *GGP* | 5 |
| 8 | L-galactose-1-phosphate phosphatase | *GPP* | 4 |
| 9 | L-galactose dehydrogenase | *GalDH* | 1 |
| 10 | L-galactono-1,4-lactone dehydrogenase | *GLDH* | 4 |
| 11 | Pectin methylesterase | *PME* | 67 |
| 12 | Pectin lyase | *PL* | 22 |
| 13 | Pectin galacturonase | *PG* | 23 |
| 14 | D-galacturonate reductase GalUR | *GalUR* | 21 |
| 15 | Myo-inositol oxygenase | *MIOX* | 8 |
| 16 | Gulono-1,4-lactone dehydrogenase | *GulLDH* | 4 |
| **Ascorbate regeneration pathway** | | | |
| 17 | Ascorbate oxidase | *AO* | 11 |

| | | | |
|---|---|---|---|
| 18 | Ascorbate peroxidase | *APX* | 8 |
| 19 | Dehydroascorbate reductase | *DHAR* | 3 |
| 20 | Mono-dehydroascorbate reductase | *MDHAR* | 8 |

**Supplementary Table 16. MSA genes involved in Glutathione metabolism and the ascorbate-glutathione pathway**

| S.No. | Name | Symbol | Function |
|---|---|---|---|
| 1 | Glutathione peroxidase | *Gpx* | Plant glutathione peroxidases (GPXs) play an important role in Reactive Oxygen Species (ROS) scavenging by catalyzing the reduction of $H_2O_2$ and other organic hydroperoxides to protect plant cells from oxidative stress damage. |
| 2 | Glutamate-cysteine ligase | *GCL* | Glutamatecysteine ligase (GCL) catalyzes the first step in glutathione biosynthesis and plays an important role in regulating the intracellular redox environment. |
| 3 | Glucose-6-phosphate 1-dehydrogenase | *G6PD* | G6PDH would increase NADPH levels stimulating the NR-dependent nitric oxide production, thus enhancing the activities of antioxidant pathways, in order to scavenge the ROS induced by salt stress |
| 4 | L-ascorbate peroxidase | *APX* | Role in ROS detoxification |
| 5 | L-ascorbate oxidase | *AO* | Role in ROS detoxification |

**Supplementary Table 17. Genes involved in the Flavonoid biosynthesis pathway with copy number of their gene family**

| S.No. | Name | Symbol | Copy number |
|---|---|---|---|
| 1 | Phenylalanine ammonia lyase | *PAL* | 11 |
| 2 | Cinnamate 4-hydroxylase | *C4H* | 3 |
| 3 | 4-coumaroyl CoA ligase | *4CL* | 19 |
| 4 | Chalcone synthase | *CHS* | 9 |
| 5 | Chalcone isomerase | *CHI* | 2 |
| 6 | Flavanone 3-hydroxylase | *F3H* | 49 |
| 7 | Isoflavone synthase | *IFS* | 54 |

| | | | |
|---|---|---|---|
| 8 | Flavonol 3′5′ -hydroxylase | *F3′5′ H* | 54 |
| 9 | Flavonol synthase | *FLS* | 49 |
| 10 | Dihydroflavonol 4-reductase | *DFR* | 35 |
| 11 | Leucoanthocyanidin dioxygenase/ anthocyanidin synthase | *LDOX* | 49 |
| 12 | Uridine diphosphate (UDP)-glucose flavonoid-3-O-glycosyltransferase | *UFGT* | 4 |
| 13 | Leucoanthocyanidin reductase | *LAR* | 28 |
| 14 | Flavone synthase | *FS* | 54 |
| 15 | Anthocyanidin reductase | *ANR* | 35 |

**Supplementary Table 18. Genes involved in the Lignin biosynthesis pathway with copy number of their gene family**

| S.No. | Name | Symbol | Copy number |
|---|---|---|---|
| 1 | Phenylalanine ammonia lyase | *PAL* | 11 |
| 2 | trans-cinnamate 4-monooxygenase | *C4H* | 3 |
| 3 | 4-coumarate--CoA ligase | *4CL* | 19 |
| 4 | Shikimate O-hydroxycinnamoyltransferase | *HCT* | 65 |
| 5 | 5-O-(4-coumaroyl)-D-quinate 3'-monooxygenase | *C3H* | 54 |
| 6 | Caffeoylshikimate esterase | *CSE* | 21 |
| 7 | Caffeic acid 3-O-methyltransferase | *COMT* | 39 |
| 8 | Caffeoyl-CoA O-methyltransferase | *CCoAOMT* | 16 |
| 9 | Cinnamoyl-CoA reductase | *CCR* | 36 |
| 10 | Ferulate-5-hydroxylase | *F5H* | NA |
| 11 | Cinnamyl-alcohol dehydrogenase | *CAD* | 36 |
| 12 | Peroxidase | *POD* | 58 |
| 13 | Laccase | *LAC* | 70 |

**Supplementary Table 20. MSA genes involved in ROS regulation and detoxification**

| S.No. | Name | Symbol | Function |
|---|---|---|---|
| 1 | L-ascorbate oxidase | *AO* | ROS detoxification |
| 2 | L-ascorbate peroxidase | *APX* | ROS detoxification |
| 3 | Aconitate hydratase | *ACO, acnA* | ROS regulation through enzyme regulation |
| 4 | Cysteine synthase | *cysK* | Maintaining ROS homeostasis |
| 5 | Glutamate--cysteine ligase | *gshA* | Antioxidant biosynthesis |
| 6 | Flavonoid 3',5'-hydroxylase | *CYP75A* | roles of flavonoids as ROS scavengers |
| 7 | Glutathione peroxidase | *gpx, btuE, bsaA* | Role as ROS scavenger |
| 8 | Molybdenum cofactor sulfurtransferase | *ABA3* | Antioxidant accumulation |

| | | | |
|---|---|---|---|
| 9 | Glyceraldehyde 3-phosphate dehydrogenase (phosphorylating) | *GAPDH, gapA* | ROS induction |
| 10 | Hexokinase | *HK* | Antioxidant accumulation |
| 11 | Frataxin | *FXN, fh* | Activation of Fe-S cluster proteins |
| 12 | Magnesium chelatase subunit H | *chlH, bchH* | Mg-chelatase in producing photosynthetic apparatus in chlorophyll biosynthesis and ROS regulation |
| 13 | Magnesium chelatase subunit D | *chlD, bchD* | Mg-chelatase in producing photosynthetic apparatus in chlorophyll biosynthesis and ROS regulation |
| 14 | Hydroxymethylbilane synthase | *hemC, HMBS* | In porphyrin biosynthesis and ROS regulation |
| 15 | 26S proteasome regulatory subunit T2 | *PSMC1, RPT2* | ROS induction |
| 16 | 20S proteasome subunit alpha 1 | *PSMA6* | Protein degradation of oxidized proteins |
| 17 | Nucleoredoxin | *NXN* | Protecting antioxidant enzymes |
| 18 | Glucose-6-phosphate 1-dehydrogenase | *G6PD, zwf* | Antioxidant biosynthesis |

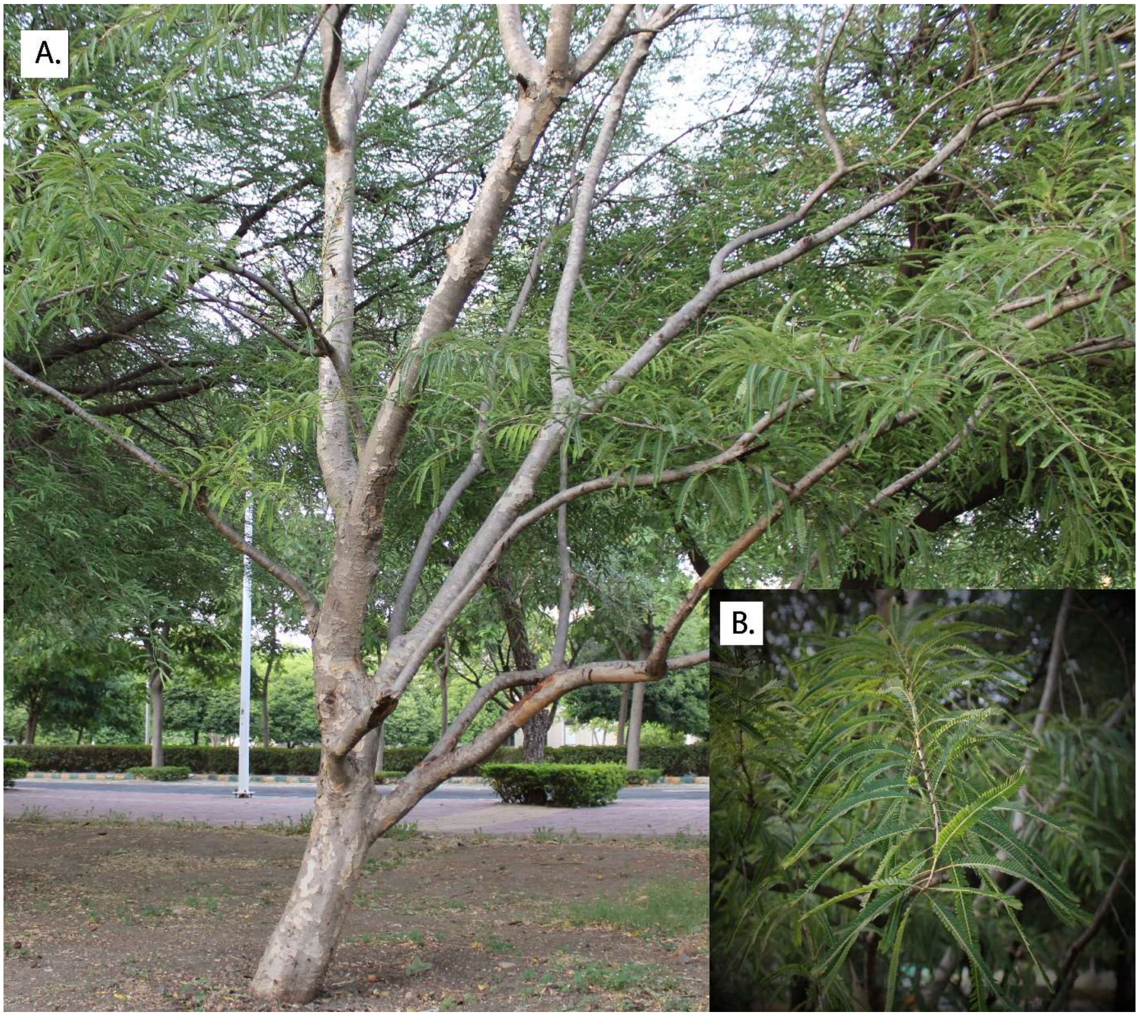


**Supplementary Figure 1. Amla plant and leaves** A. The whole plant of *P. emblica* located inside the campus of Indian Institute of Science Education and Research Bhopal at Bhopal, India (23.2858° N, 77.2755° E) that was used for genome sequencing in this study. B. The closer image of *P. emblica* leaves.

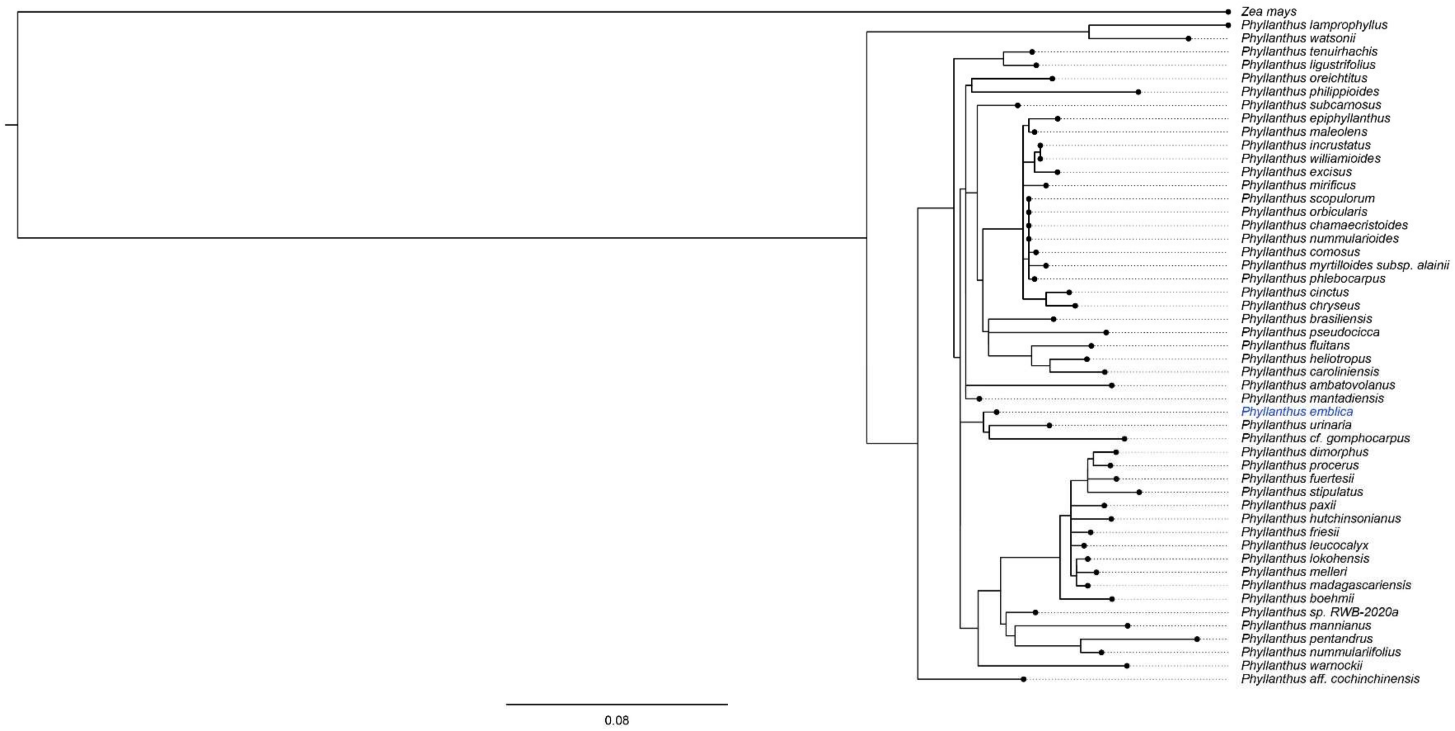


**Supplementary Figure 2. Phylogenetic tree of 50 *Phyllanthus* species and *Zea mays* as an outgroup using Maturase K (*MatK*) sequences**

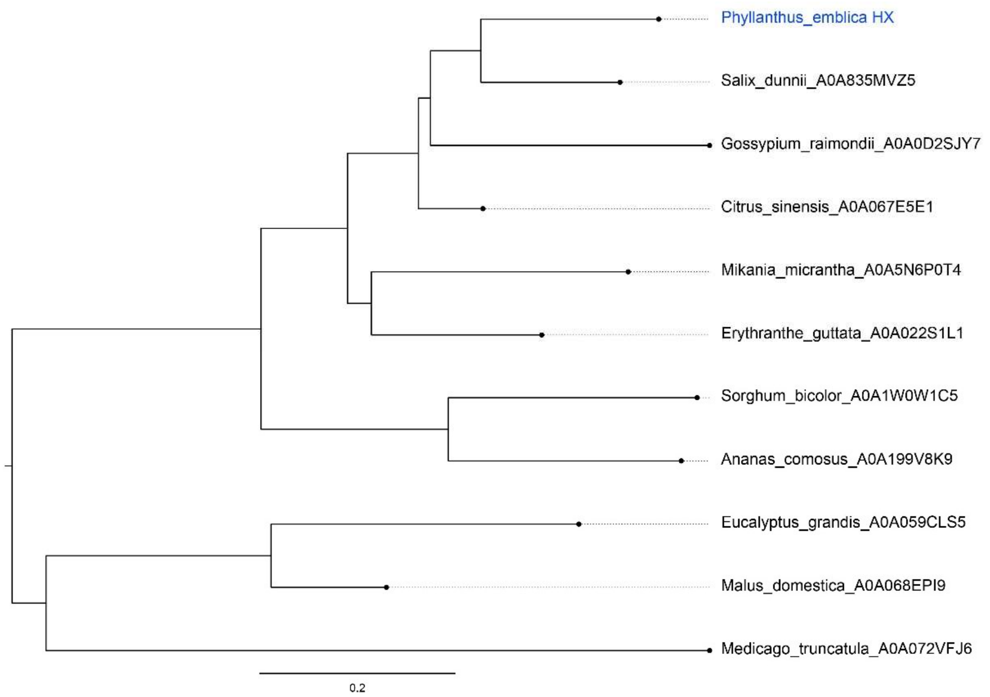


**Supplementary Figure 3. Phylogeny of Hexokinase (*HX*) gene of Ascorbate biosynthesis pathway**

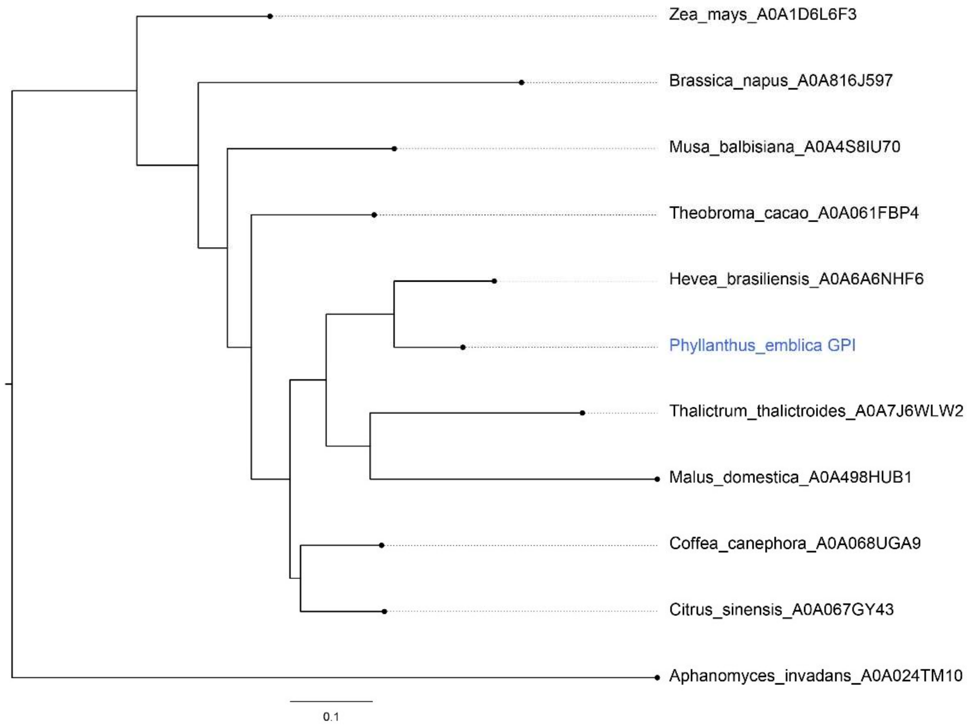


**Supplementary Figure 4. Phylogeny of Glucose 6-phosphate isomerase (*GPI*) gene of Ascorbate biosynthesis pathway**

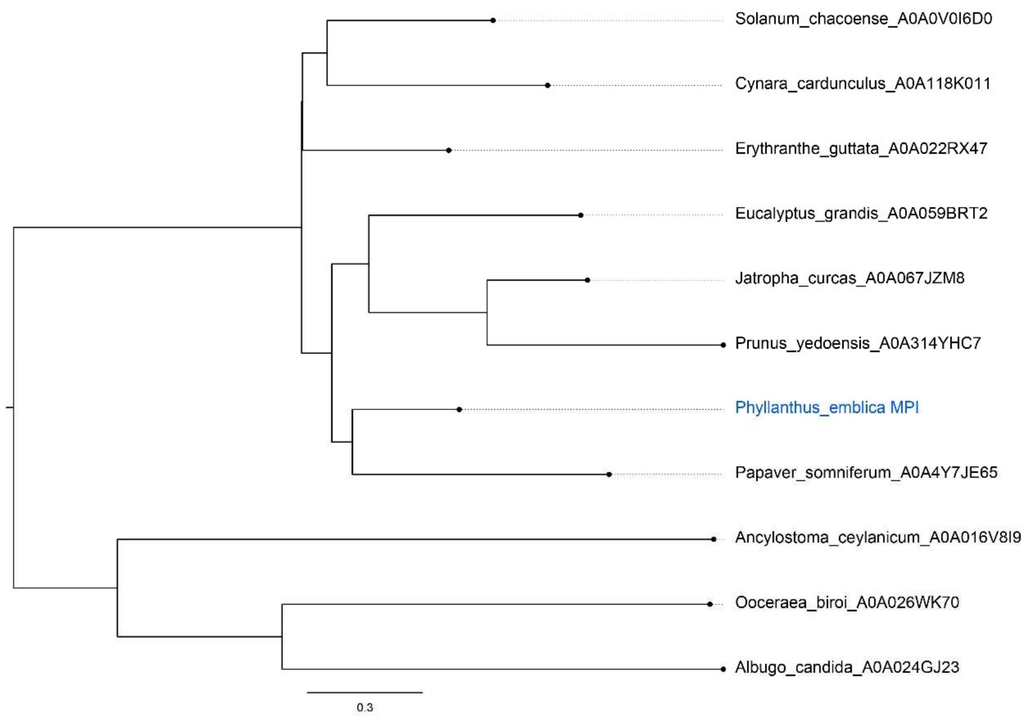


**Supplementary Figure 5. Phylogeny of Mannose6-Phosphate isomerase (*MPI*) gene of Ascorbate biosynthesis pathway**

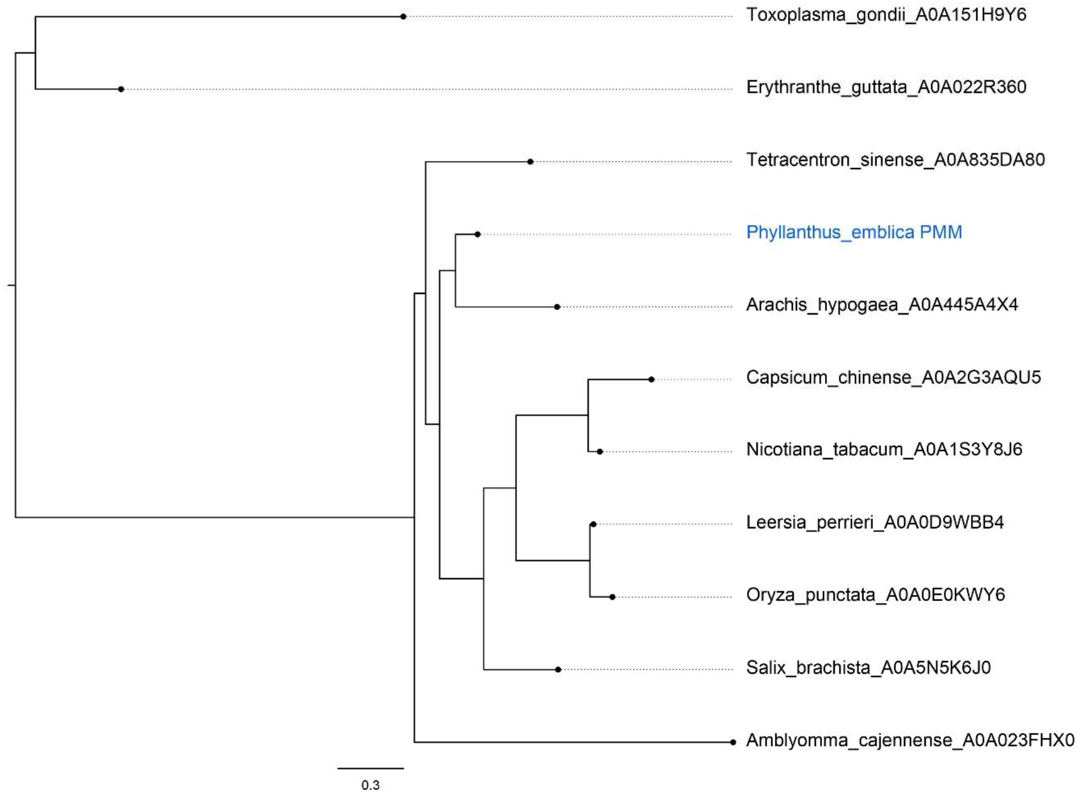


**Supplementary Figure 6. Phylogeny of Phosphomannomutase (*PMM*) gene of Ascorbate biosynthesis pathway**

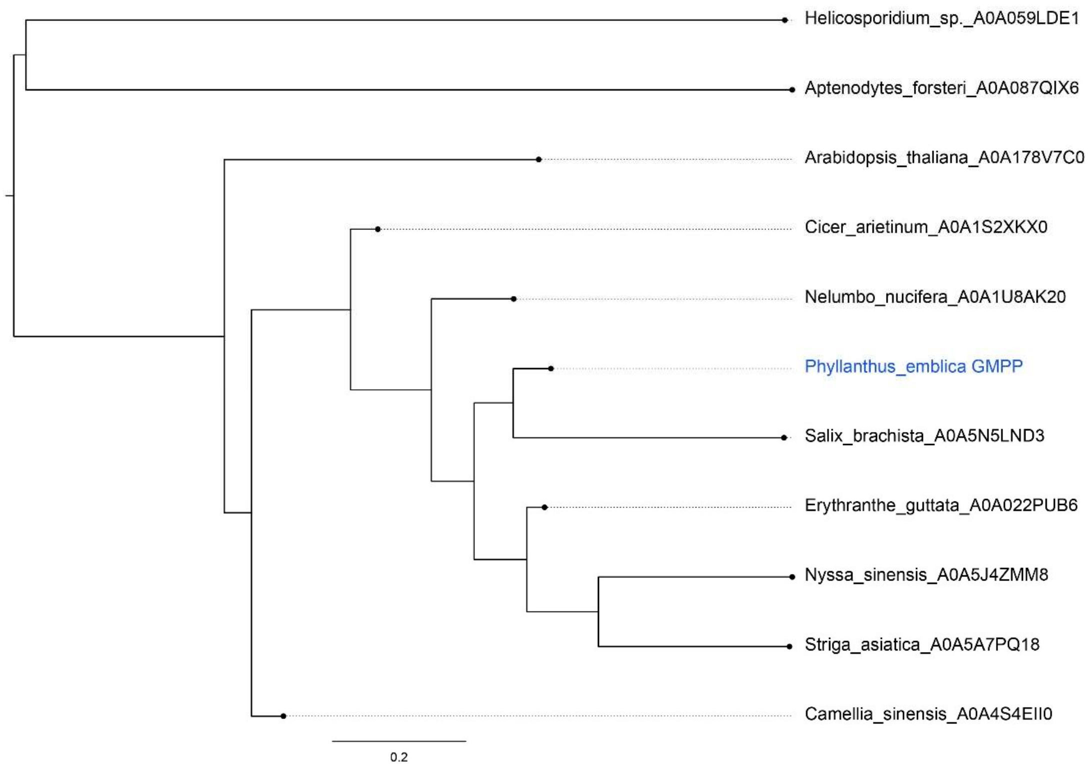


**Supplementary Figure 7. Phylogeny of GDP-mannose pyrophosphorylase (*GMPP*) gene of Ascorbate biosynthesis pathway**

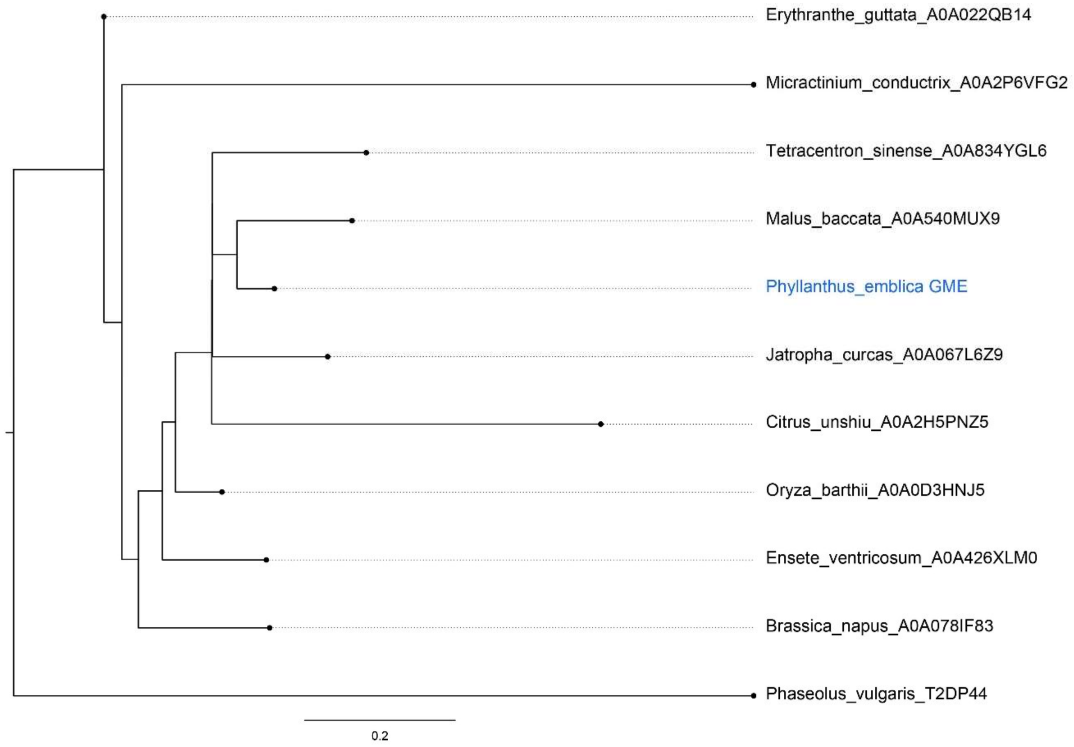


**Supplementary Figure 8. Phylogeny of GDP-D-Mannose 3',5'-epimerase (*GME*) gene of Ascorbate biosynthesis pathway**

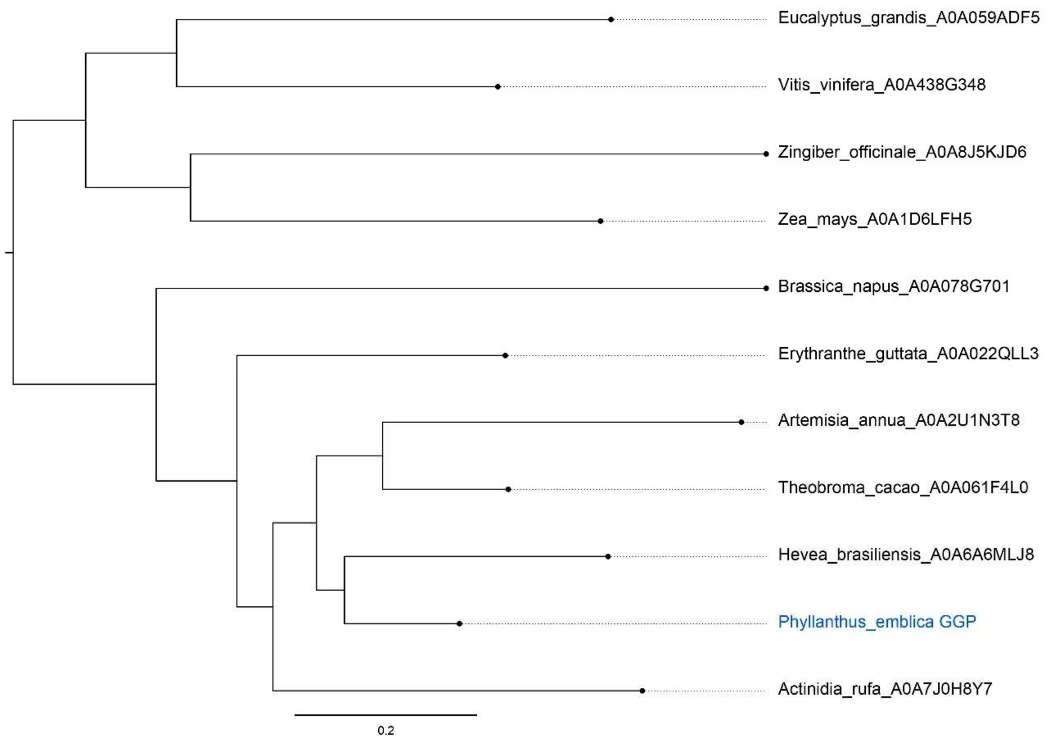


**Supplementary Figure 9. Phylogeny of GDP-L-galactose phosphorylase (*GGP*) gene of Ascorbate biosynthesis pathway**

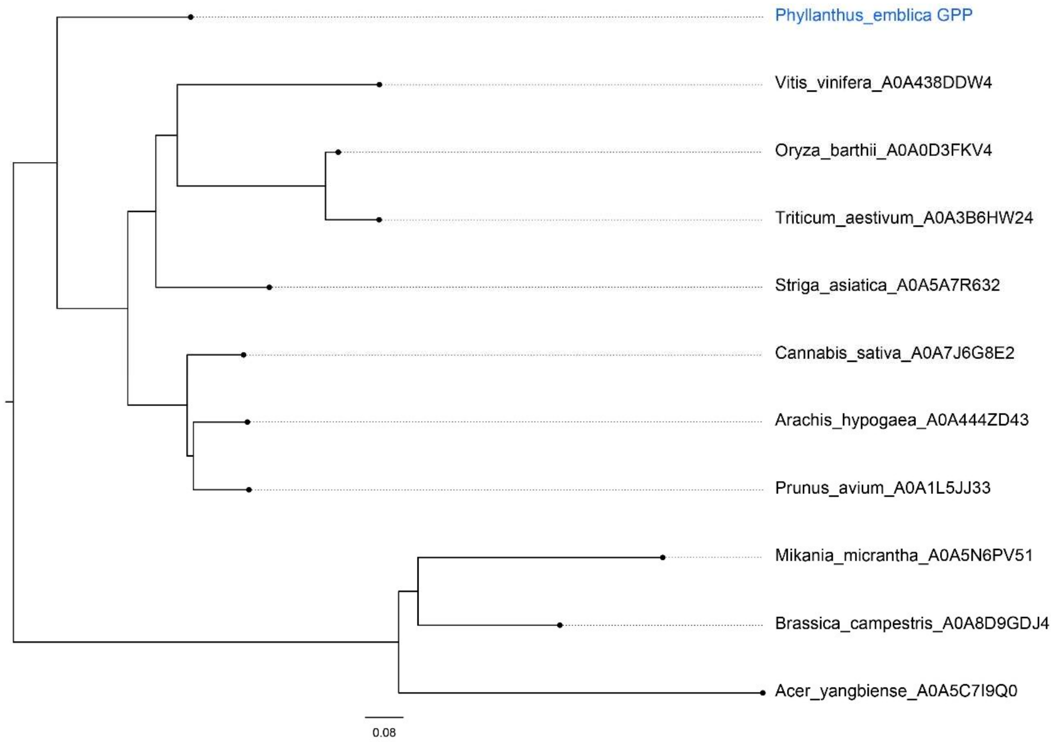


**Supplementary Figure 10. Phylogeny of L-galactose-1-phosphate phosphatase (*GPP*) gene of Ascorbate biosynthesis pathway**

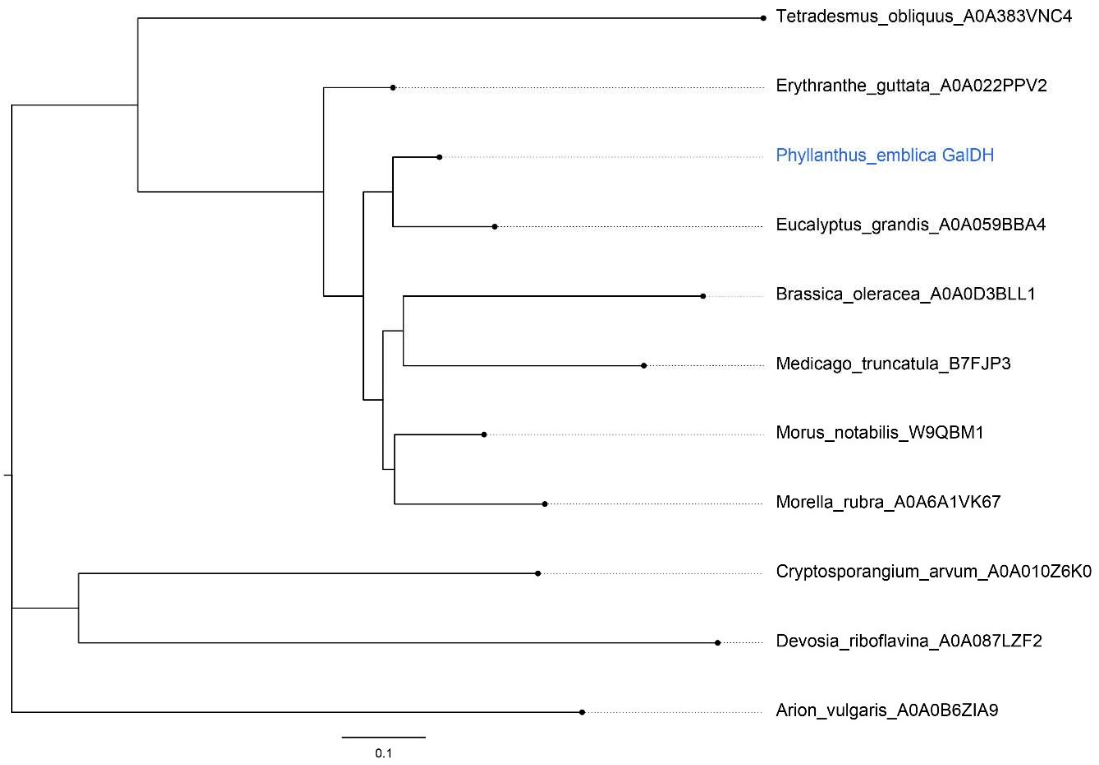


**Supplementary Figure 11. Phylogeny of L-galactose dehydrogenase (*GalDH*) gene of Ascorbate biosynthesis pathway**

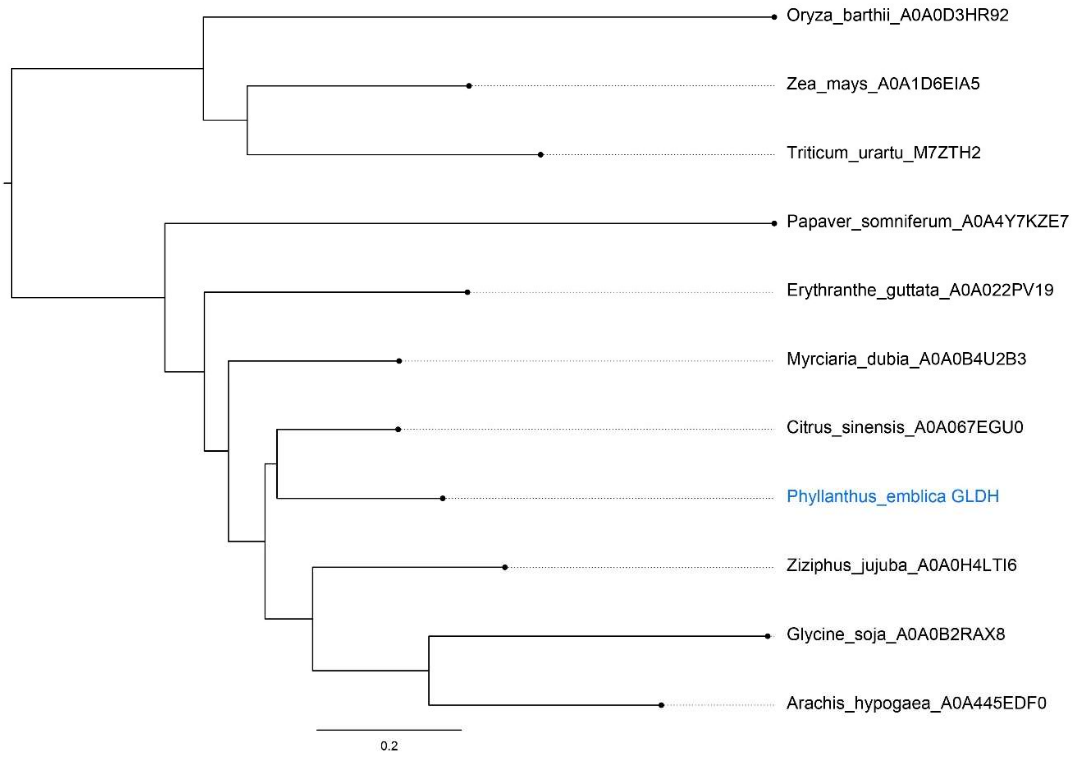


**Supplementary Figure 12. Phylogeny of L-galactono-1,4-lactone dehydrogenase (*GLDH*) gene of Ascorbate biosynthesis pathway**

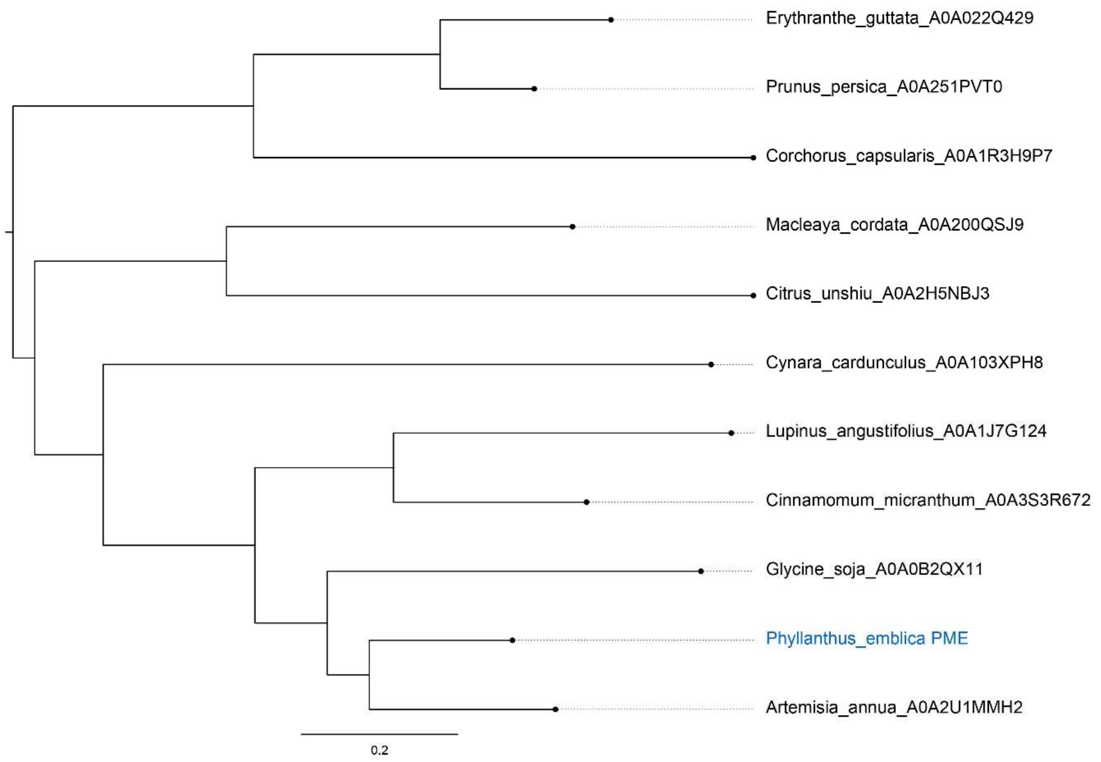


**Supplementary Figure 13. Phylogeny of Pectin methylesterase (*PME*) gene of Ascorbate biosynthesis pathway**

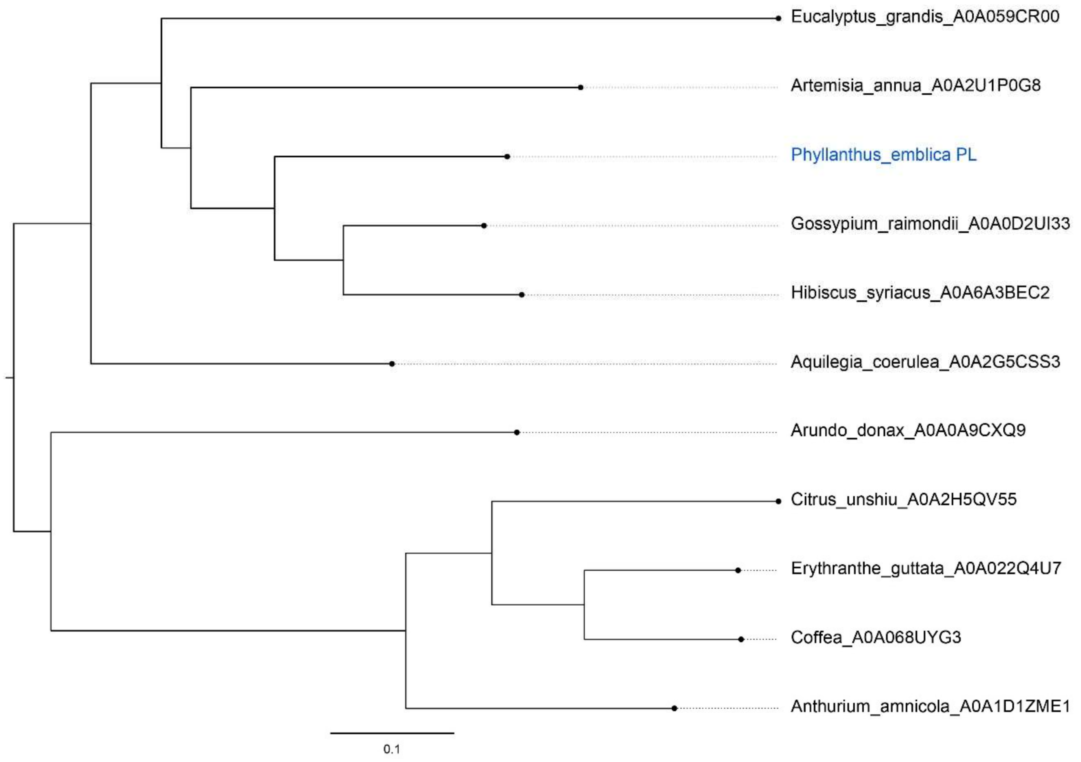


**Supplementary Figure 14. Phylogeny of Pectin lyase (*PL*) gene of Ascorbate biosynthesis pathway**

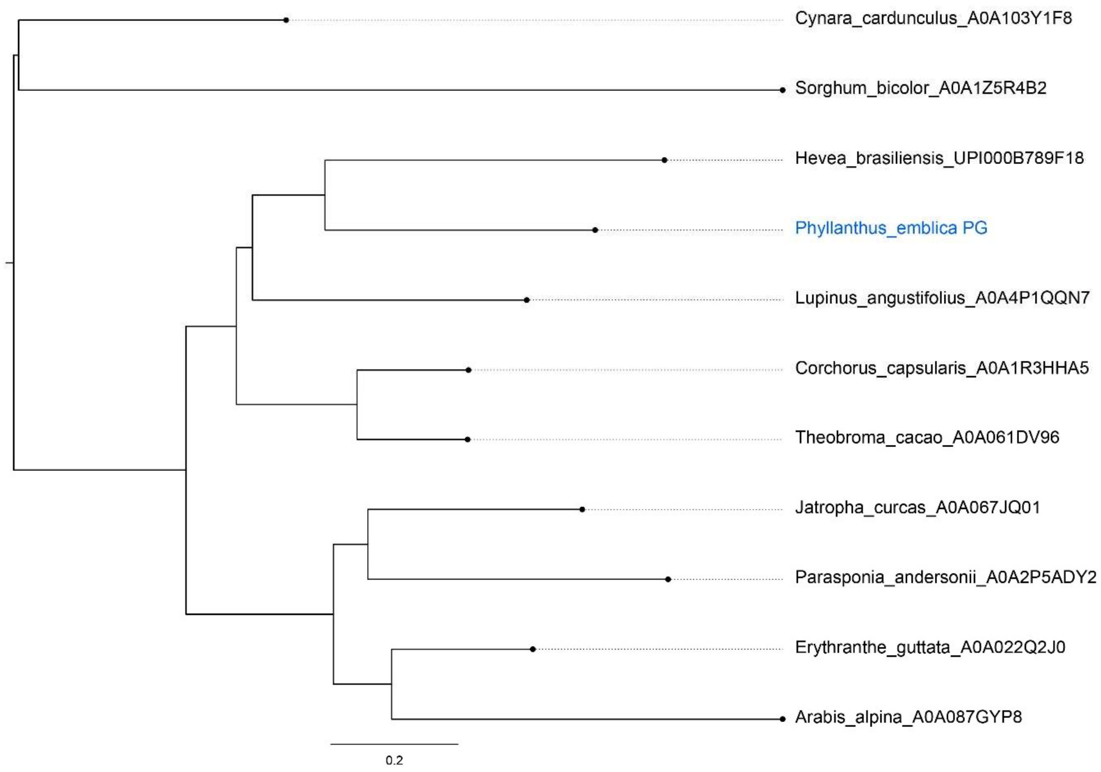


**Supplementary Figure 15. Phylogeny of Polygalacturonase (*PG*) gene of Ascorbate biosynthesis pathway**

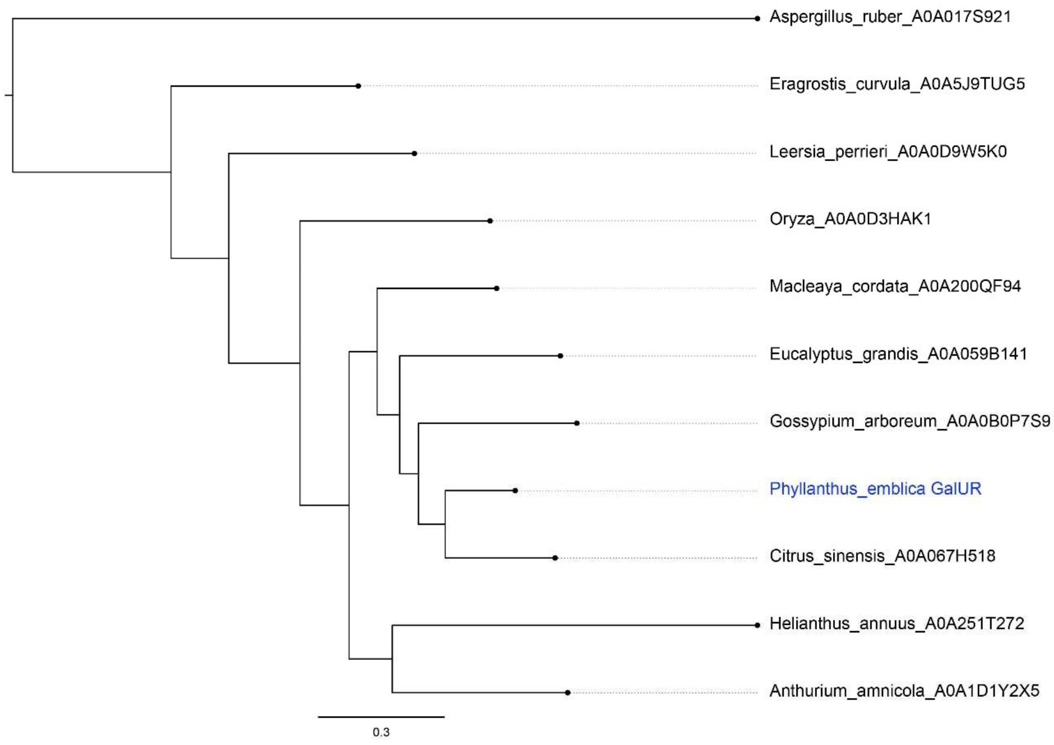


**Supplementary Figure 16. Phylogeny of D-galacturonate reductase (*GalUR*) gene of Ascorbate biosynthesis pathway**

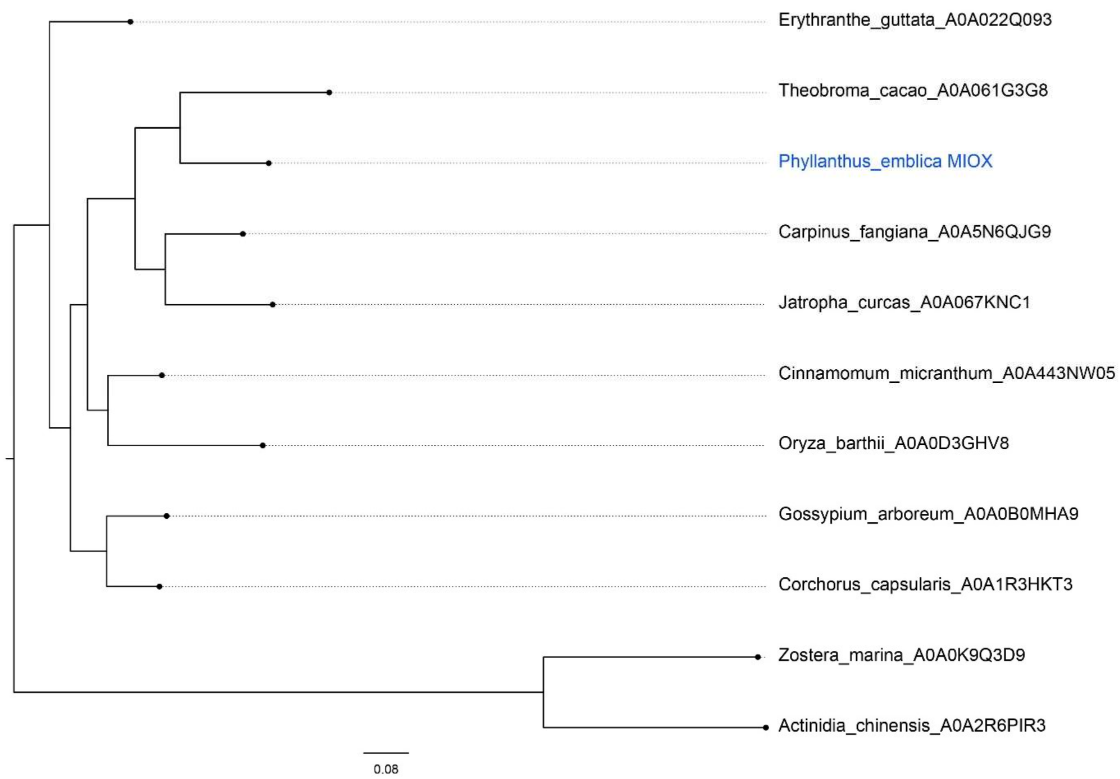


**Supplementary Figure 17. Phylogeny of Myo-inositol oxygenase (*MIOX*) gene of Ascorbate biosynthesis pathway**

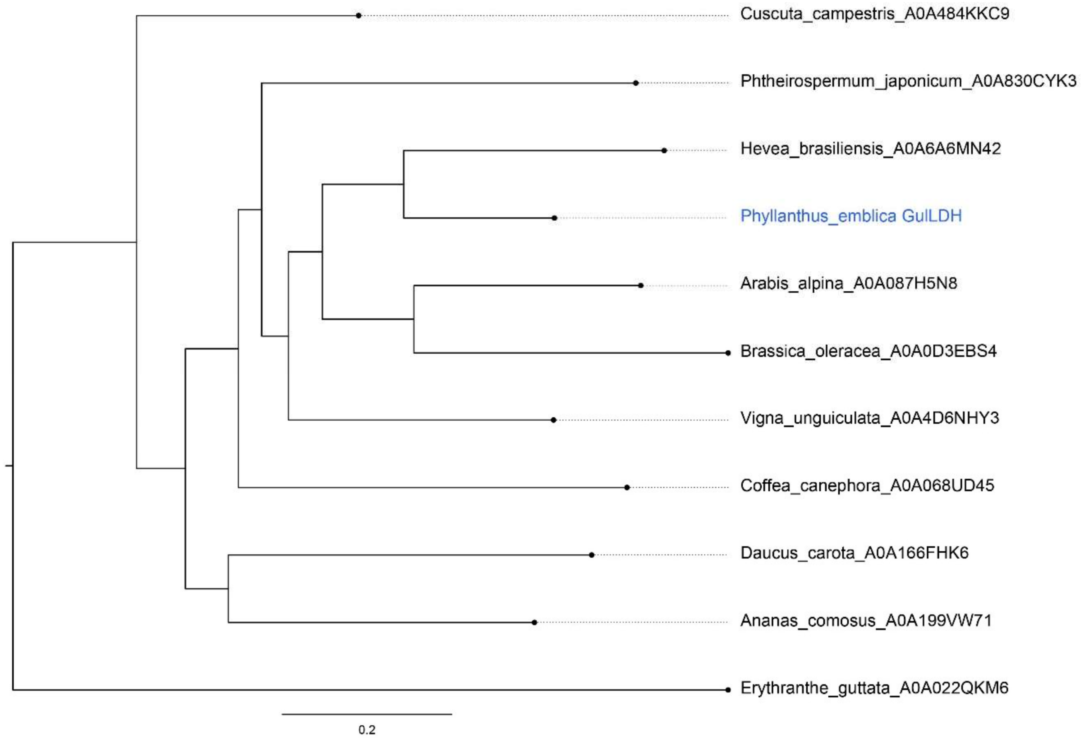


**Supplementary Figure 18. Phylogeny of Gulono-1,4-lactone dehydrogenase (*GulLDH*) gene of Ascorbate biosynthesis pathway**

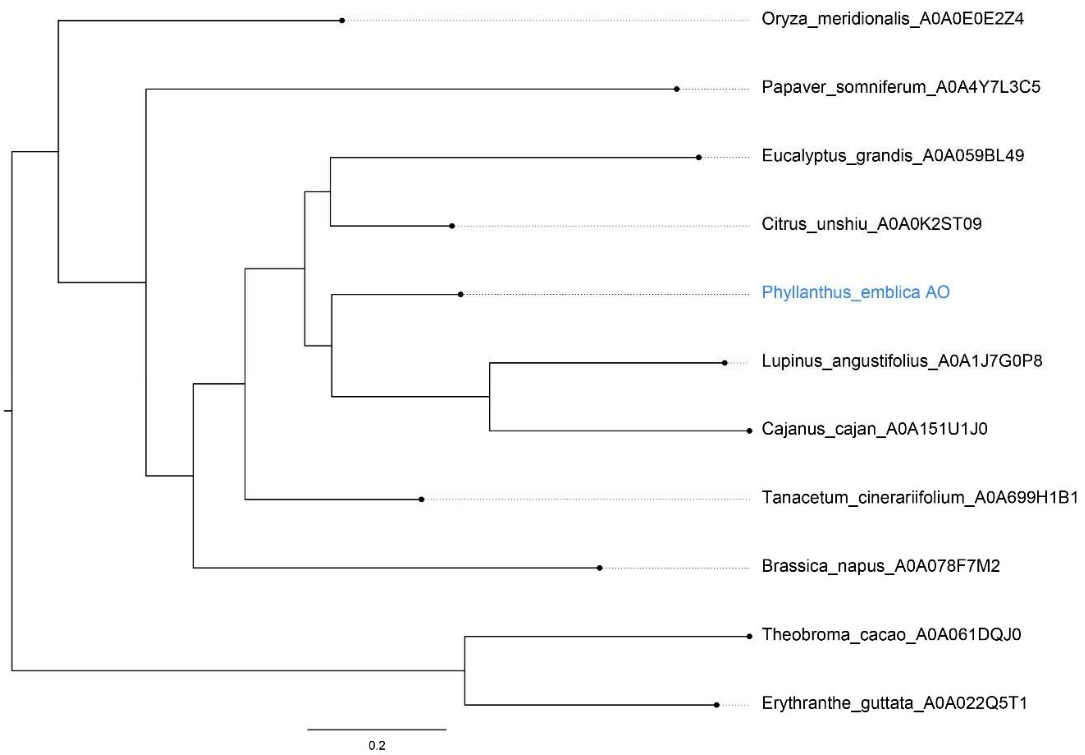


**Supplementary Figure 19. Phylogeny of Ascorbate oxidase (*AO*) gene of Ascorbate regeneration pathway**

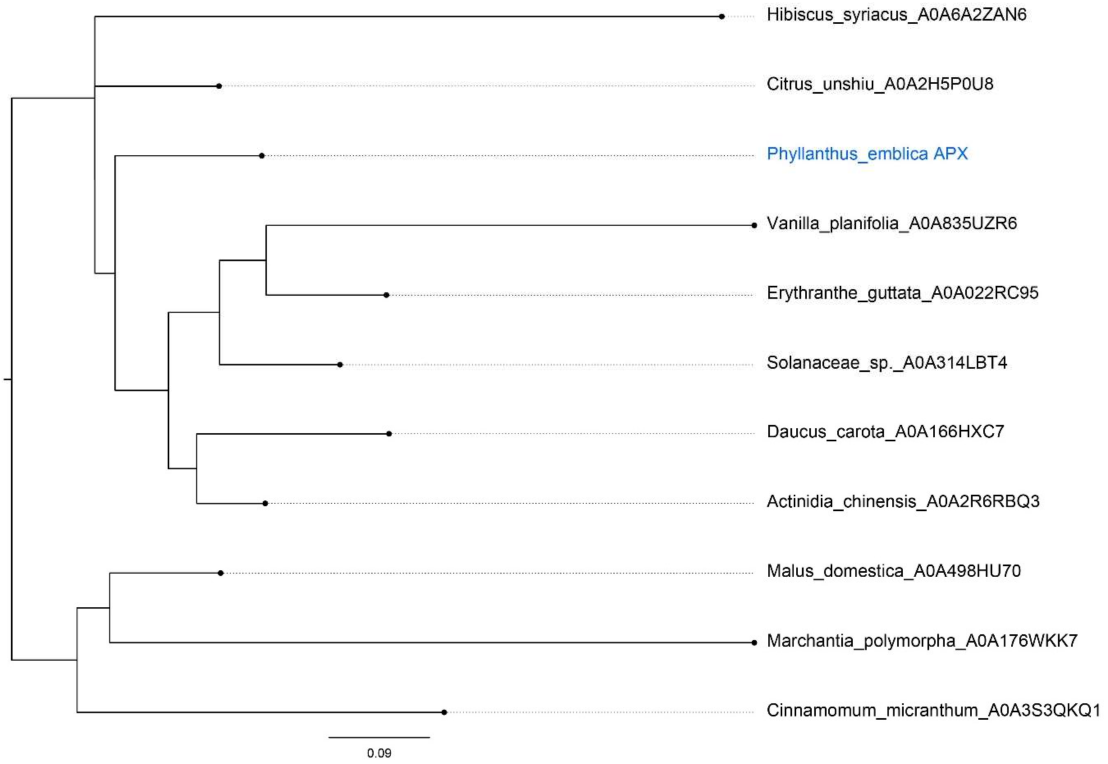


**Supplementary Figure 20. Phylogeny of Ascorbate peroxidase (*APX*) gene of Ascorbate regeneration pathway**

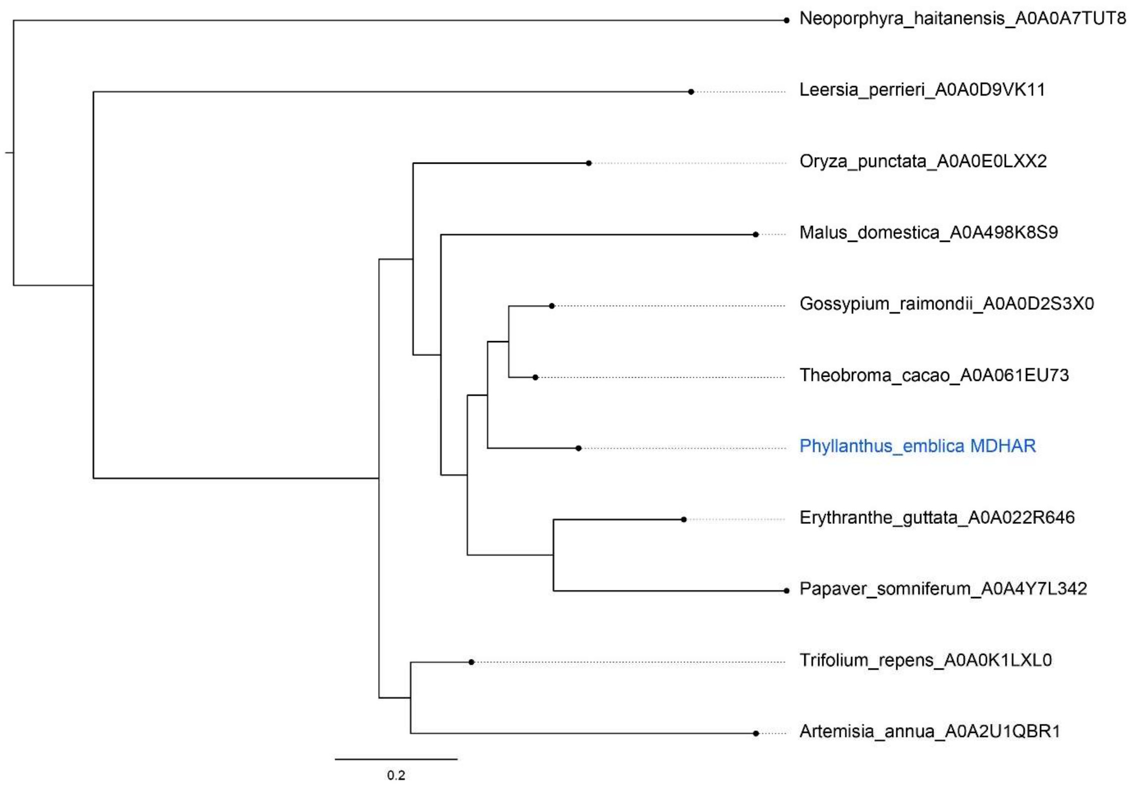


**Supplementary Figure 21. Phylogeny of Monodehydroascorbate reductase (*MDHAR*) gene of Ascorbate regeneration pathway**

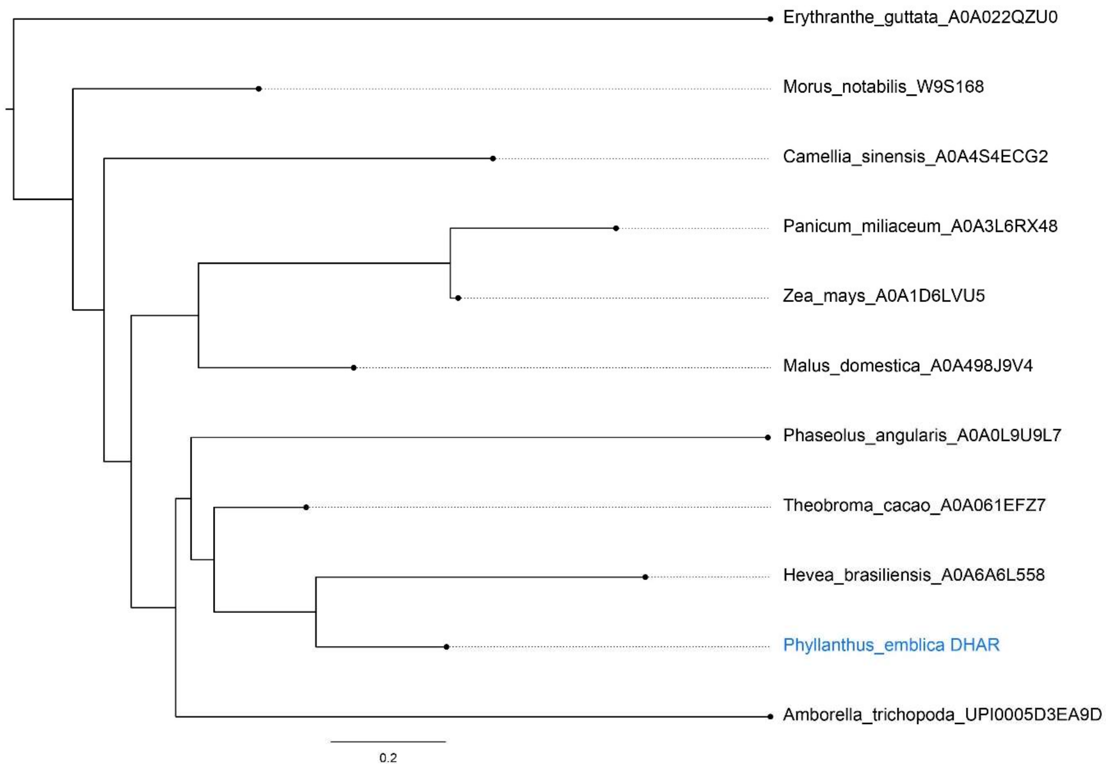


**Supplementary Figure 22. Phylogeny of Dehydroascorbate reductase (*DHAR*) gene of Ascorbate regeneration pathway**

**References:**


Altschul, S. F., W. Gish, et al. (1990). "Basic local alignment search tool." Journal of molecular biology 215(3): 403-410.

Chakraborty, A., S. Mahajan, et al. (2022). "Genome sequencing and comparative analysis of Ficus benghalensis and Ficus religiosa species reveal evolutionary mechanisms of longevity." Iscience: 105100.

Chakraborty, A., S. Mahajan, et al. (2021). "Genome sequencing of turmeric provides evolutionary insights into its medicinal properties." Communications biology 4(1): 1-12.

Group, A. P. (2009). "An update of the Angiosperm Phylogeny Group classification for the orders and families of flowering plants: APG III." Botanical Journal of the Linnean Society 161(2): 105-121.

Ha, J., S. Shim, et al. (2019). "Genome sequence of Jatropha curcas L., a non-edible biodiesel plant, provides a resource to improve seed-related traits." Plant biotechnology journal 17(2): 517-530.

Inglis, P. W., L. R. Mata, et al. (2018). "DNA barcoding for the identification of Phyllanthus taxa used medicinally in Brazil." Planta medica 84(17): 1300-1310.

Jaiswal, S. K., S. Mahajan, et al. (2021). "The genome sequence of Aloe vera reveals adaptive evolution of drought tolerance mechanisms." Iscience 24(2): 102079.

Kawakita, A. and M. Kato (2017). Diversity of Phyllanthaceae plants. Obligate pollination mutualism, Springer: 81-115.

Kumar, A. and K. Singh (2012). "Isolation of high quality RNA from Phyllanthus emblica and its evaluation by downstream applications." Molecular biotechnology 52(3): 269-275.

Liu, X., H. Ma, et al. (2018). "Development of novel EST-SSR markers for Phyllanthus emblica (Phyllanthaceae) and cross-amplification in two related species." Applications in Plant Sciences 6(7): e01169.

Mahajan, S., A. Chakraborty, et al. (2021). "Genome sequencing and assembly of Tinospora cordifolia (Giloy) plant." BioRxiv.

Wang, M., Z. Gu, et al. (2021). "High-quality genome assembly of an important biodiesel plant, Euphorbia lathyris L." DNA Research 28(6): dsab022.

Xi, Z., B. R. Ruhfel, et al. (2012). "Phylogenomics and a posteriori data partitioning resolve the Cretaceous angiosperm radiation Malpighiales." Proceedings of the National Academy of Sciences 109(43): 17519-17524.

Xu, W., D. Wu, et al. (2021). "Genomic insights into the origin, domestication and genetic basis of agronomic traits of castor bean." Genome biology 22(1): 1-27.
